# Bacterial cancer therapy in autochthonous colorectal cancer affects tumor growth and metabolic landscape

**DOI:** 10.1172/jci.insight.139900

**Published:** 2021-10-28

**Authors:** Gillian M. Mackie, Alastair Copland, Masumi Takahashi, Yumiko Nakanishi, Isabel Everard, Tamotsu Kato, Hirotsugu Oda, Takashi Kanaya, Hiroshi Ohno, Kendle M. Maslowski

**Affiliations:** 1 University of Birmingham, Institute of Immunology and Immunotherapy and Institute of Metabolism and Systems Research, Birmingham, United Kingdom.; 2 Laboratory for Intestinal Ecosystem, RIKEN Institute for Integrative Medical Science, Yokohama, Japan.; 3 Intestinal Microbiota Project, Kanagawa Institute of Industrial Science and Technology, Kawasaki, Japan.; 4 Immunobiology Laboratory, Graduate School of Medical Life Science, Yokohama City University, Yokohama, Japan.; 5 Laboratory for Integrative Genomics, RIKEN Center for Integrative Medical Sciences, Yokohama, Japan.; 6 Inflammatory Disease Section, National Human Genome Research Institute, NIH, Bethesda, Maryland, USA.

**Keywords:** Gastroenterology, Bacterial infections, Colorectal cancer

## Abstract

Bacterial cancer therapy (BCT) shows great promise for treatment of solid tumors, yet basic mechanisms of bacterial-induced tumor suppression remain undefined. Attenuated strains of *Salmonella enterica* serovar Typhimurium (*S*Tm) have commonly been used in mouse models of BCT in xenograft and orthotopic transplant cancer models. We aimed to better understand the tumor epithelium–targeted mechanisms of BCT by using autochthonous mouse models of intestinal cancer and tumor organoid cultures to assess the effectiveness and consequences of oral treatment with aromatase A–deficient *S*Tm (*STm*^*Δ*^*aroA*). *STm*^*Δ*^*aroA* delivered by oral gavage significantly reduced tumor burden and tumor load in both a colitis-associated colorectal cancer (CAC) model and in a spontaneous *Apc^min/+^* intestinal cancer model. *STm*^*Δ*^*aroA* colonization of tumors caused alterations in transcription of mRNAs associated with tumor stemness, epithelial-mesenchymal transition, and cell cycle. Metabolomic analysis of tumors demonstrated alteration in the metabolic environment of *STm*^*Δ*^*aroA*-treated tumors, suggesting that *STm*^*Δ*^*aroA* imposes metabolic competition on the tumor. Use of tumor organoid cultures in vitro recapitulated effects seen on tumor stemness, mesenchymal markers, and altered metabolome. Furthermore, live *STm*^*Δ*^*aroA* was required, demonstrating active mechanisms including metabolite usage. We have demonstrated that oral BCT is efficacious in autochthonous intestinal cancer models, that BCT imposes metabolic competition, and that BCT has direct effects on the tumor epithelium affecting tumor stem cells.

## Graphical abstract

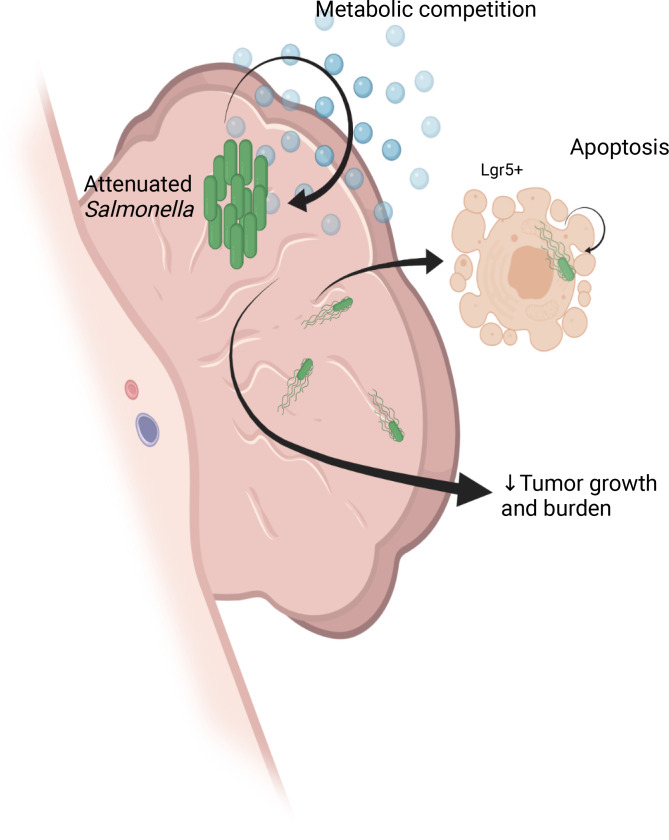

## Introduction

The use of bacteria as cancer therapeutics (bacterial cancer therapy [BCT]) dates back to the late 1800s, when the field of BCT was initiated by William Coley (1). Prior observations of spontaneous tumor regression following infection of patients’ tumors had led Coley to treat cancer patients with intratumoral injections of bacterial preparations (1). Despite this early work on BCT, there is only 1 currently in the clinic — Bacillus Calmette-Guérin vaccine (BCG) therapy for superficial bladder cancer (2).

The advancement of molecular genetics has enabled bacteria to be effectively attenuated to remove adverse effects and has been engineered to deliver different payloads. Therefore, there has been a resurgence in interest in BCT in the past 20 years, with many studies showing efficacy of attenuated bacterial treatments in xenograft and orthotopic transplant tumor models, with *Salmonella enterica* serovar Typhimurium (*S*Tm) being by far the most studied (3–9). Attenuated *S*Tm are extremely tumor tropic; a therapy that can give such tumor-tissue selectivity is very desirable and enables further engineering to deliver drugs, immune adjuvants, or other antitumor agents (3). Despite this interest, very little is understood about the underlying mechanisms of BCT-mediated tumor suppression, which is hampering its practical application.

Given that BCG is delivered directly to the bladder epithelium, and that direct interaction is necessary for direct cytotoxic effects (2, 10), we hypothesized that other BCT can exert direct effects on tumor cells. To date, there have been limited BCT studies using autochthonous models of cancer. Studies on BCT thus far have utilized xenograft or orthotopic transplant models of cancer, often in immunocompromised mice, and these may not fully model complex disease in patients and therefore may not be entirely predictive of efficacy or mechanism of BCT. One study using an autochthonous model of prostate cancer showed limited efficacy of a heavily attenuated *S*Tm strain (CRC2631) when injected i.p. on a weekly basis (11).

Since intestinal innate immune pathways protect from both *S*Tm infection and tumorigenesis (12, 13), we aimed to determine if *S*Tm treatment could effectively treat autochthonous tumors of the intestine. We reasoned that intestinal cancer would be an appropriate target for BCT using *S*Tm, as the natural route of infection is via the intestine and, therefore, would likely enable better invasion and interaction between the bacteria and the tumor, which would overcome issues of poor dissemination of orally administered *S*Tm to tumors at nongastrointestinal sites (14). Treating colorectal cancer (CRC) patients by oral delivery of attenuated *S*Tm is feasible since oral vaccines for *S*. typhi are widely used and tolerated (15). Oral delivery of *S*Tm may also avoid problems of tumor homing and toxicity that have been observed when delivering *S*Tm i.v. to patients (16).

We utilized *S*Tm deficient for aromatase A (*STmΔaroA*) (UF020; ref. 17) to assess whether BCT could be effective for treating mouse models of colorectal cancer. Aromatase A–deficient *S*Tm are auxotrophic for aromatic amino acids (AAs). The tumor microenvironment is often enriched with AAs (18, 19), which may aid its specific colonization of tumor tissue compared with normal intestine where they are not freely available. *S*Tm^Δ^*aroA* is commonly used as a vaccine strain (20) and has also previously been used successfully as a BCT in tumor transplant models (14, 21–23). Using a model of colitis-associated colorectal cancer (CAC) and a spontaneous model of intestinal cancer, *Apc^min/+^* mice, we show that oral delivery of an attenuated *S*Tm potently reduces tumor burden. Transcriptomic and metabolomic analyses, coupled with use of tumor organoids in vitro, demonstrated restoration of epithelial markers by *S*Tm, including reduced tumor stem markers, and found that *S*Tm impose metabolic competition, which is likely central to antitumor effects.

## Results

### Orally administered STm^ΔaroA^ reduces gastrointestinal tumor burden.

We first determined whether orally administered *STmΔaroA* would effectively colonize intestinal polyps in the *Apc^min/+^* mouse model. These mice carry a mutation in the adenomatous polyposis coli gene (*Apc*), which results in multiple intestinal neoplasia (min), serving as a model of human familial adenomatous polyposis (FAP). In mice, the *Apc* mutation results largely in small intestinal (SI) neoplasia (100% penetrance) and not colonic neoplasia (approximately 50% penetrance with few tumors). We treated *Apc^min/+^* or littermate *Apc^+/+^* mice with oral gavage of 5 × 10^9^ CFU *STmΔaroA* and assessed bacterial burden in a range of tissues at various time points after administration. Indeed, *STmΔaroA* colonized polyps in the ileum within 4 hours of treatment, followed by a peak in number at 24 hours and a contraction by 1 week after administration. Lower levels could still be observed 2 weeks after administration (Supplemental Figure 1; supplemental material available online with this article; https://doi.org/10.1172/jci.insight.139900DS1). In contrast, there were much lower CFUs in the normal SI tissue, though showing a similar trajectory over time, and WT non–tumor-bearing mice showed even lower burden in the normal SI (Supplemental Figure 1). This is likely reflected in the fact that *Apc^min/+^* mice have extensive polyps and aberrant crypts throughout the SI. Mesenteric lymph nodes showed a gradual increase in *STmΔaroA* CFUs over 2 weeks, with slightly higher levels in tumor-bearing mice than in non–tumor-bearing mice, though these levels were far less than seen within tumors (Supplemental Figure 1). Peyer’s patches showed initial colonization at 24 hours, which decreased over time, comparable in tumor-bearing mice and non–tumor-bearing mice (Supplemental Figure 1). Analysis of spleen CFUs showed some low-level colonization in few mice (1 from each genotype) 2 weeks after administration (Supplemental Figure 1). Finally, analysis of ileal content and feces showed a surprisingly low number of CFUs. Tumor-bearing mice had greater levels in the ileal content 24 hours after administration. CFUs recovered from the feces demonstrated a delayed peak (at 72 hours compared with 24 hours) in non–tumor-bearing mice. Overall, this analysis showed that, as per previous publications (4–9), attenuated *ST*m preferentially colonize tumor tissue over normal tissues and that, within intestinal polyps, colonization decreases by 2 weeks. We therefore proceeded to assess the efficacy of *STmΔaroA* treatment in 2 models of intestinal cancer by giving weekly oral dosing.

**Figure 1:**
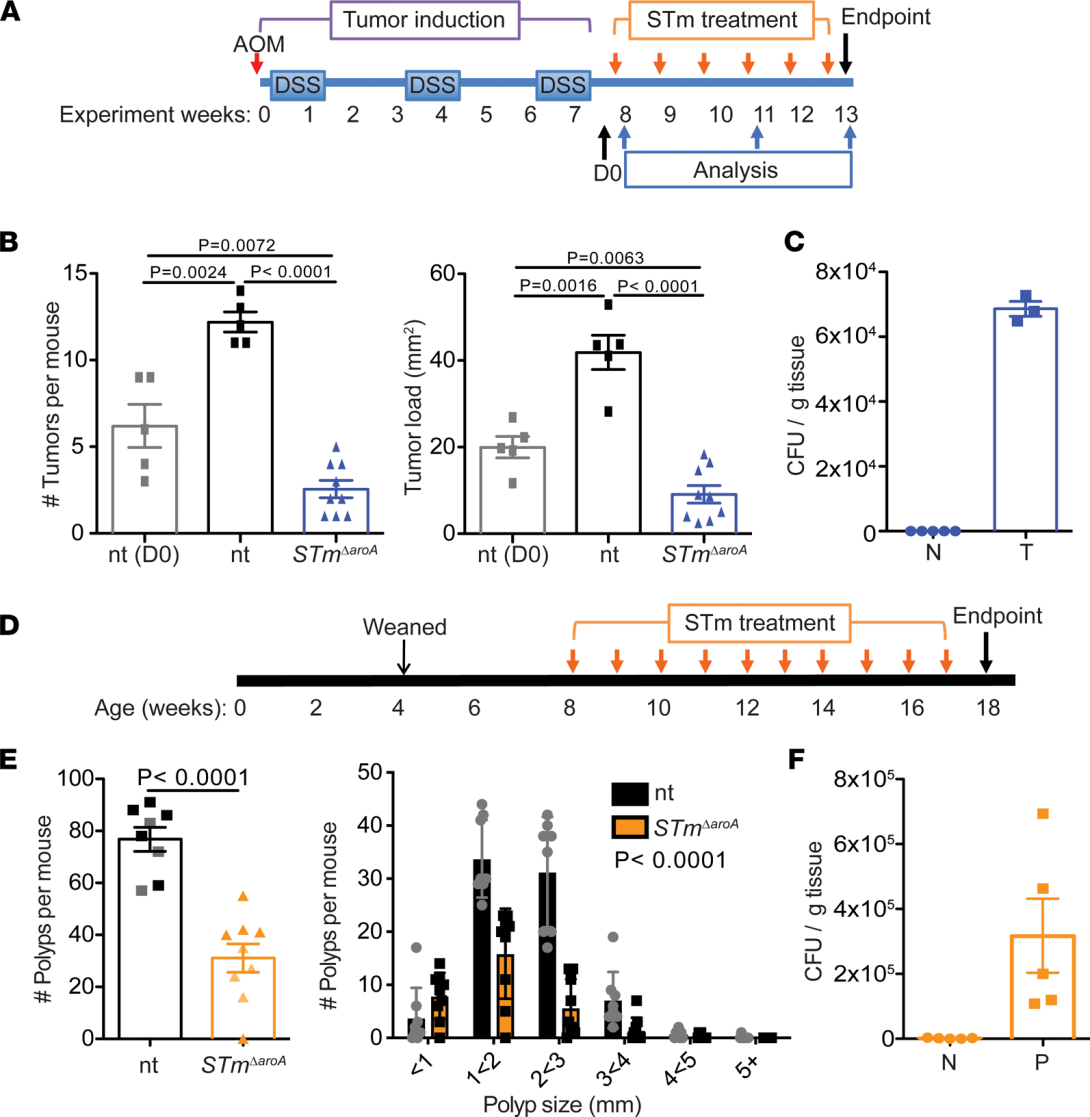
Oral delivery of attenuated *S*Tm reduces intestinal tumor burden. (**A**) Schematic of AOM/DSS-induced CAC model and *STm^ΔaroA^* treatment. (**B**) Tumor burden (number of tumors/mouse) and tumor load (cumulative tumor size per mouse, mm^2^) in nontreated (nt) and *STm^ΔaroA^*-treated mice. *n* = 5 for D0 and nt groups; *n* = 9 for *STm^ΔaroA^*-treated mice. Representative of 4 independent experiments. Female mice were used in this experiment. (**C**) CFU of *STm^ΔaroA^* in normal (N) and tumor (T) tissue from *STm^ΔaroA^*-treated mice in the CAC model. (**D**) Schematic of *Apc^min/+^* mouse *STm^ΔaroA^* treatment. (**E**) Polyp burden and polyp size per mouse in nontreated (nt) and *STm^ΔaroA^*-treated mice. Data pooled from 2 independent experiments using both male and female mice, nt *n* = 8 (4F, 4M), *STm^ΔaroA^*-treated *n* = 9 (5F, 4M). Lighter shaded mice in NT and *S*Tm indicate mice used for RNA analysis in Figure 4B. (**F**) CFU of *STm^ΔaroA^* in normal (N) and polyp (P) tissue from *STm^ΔaroA^*-treated mice in the *Apc^min/+^* model; data are shown as mean ± SD. One-way ANOVA (**B**) or 2-tailed *t* test (**E**) were used; data are shown as mean ± SD.

We induced colon tumors in C57B6/J mice using a well-described model of CAC, which has 100% penetrance (13, 24) (Figure 1A). After tumor induction, mice were split into treatment groups, ensuring equivalent colitis severity between groups. Supplemental Figure 2 shows weight loss during the azoxymethane/dextran sodium sulphate (AOM/DSS) protocol. Following recovery from the final dose of DSS (1 to 2 weeks), mice were given 5 × 10^9^ CFU *STmΔaroA*, or vehicle control (PBS), by oral gavage once per week for 6 weeks. From the start of treatment (denoted D0), mice had well-developed colonic tumors (Figure 1B). Tumor burden and tumor load was significantly decreased in *STm*^ΔaroA^-treated mice, compared with both D0 and 6-week control-treated mice (Figure 1B). This indicates that *STmΔaroA* treatment by oral delivery could reduce existing tumor burden and prevent further tumor development or growth. We measured *STmΔaroA* CFUs in tumors at the end of the protocol and could confirm colonization in the colon tumor but not normal tissue (Figure 1C).

Next, we tested *STmΔaroA* treatment in *Apc^min/+^* mice. We treated *Apc^min/+^* mice with 5 × 10^9^ CFU *STmΔaroA* by oral gavage once per week for 10 weeks, from 8 weeks of age (Figure 1D). At this age, the SI had already developed a large number of polyps and they continued to grow in size, with mice at 18 weeks showing large well-developed polyps throughout the SI tract. Treatment of *Apc^min/+^* mice with *STmΔaroA* substantially reduced both the polyp burden and size (Figure 1E). Colonization of SI polyps by *STmΔaroA* was confirmed at the end of the treatment, with no colonies observed in the normal surrounding tissue (Figure 1F).

**Figure 2:**
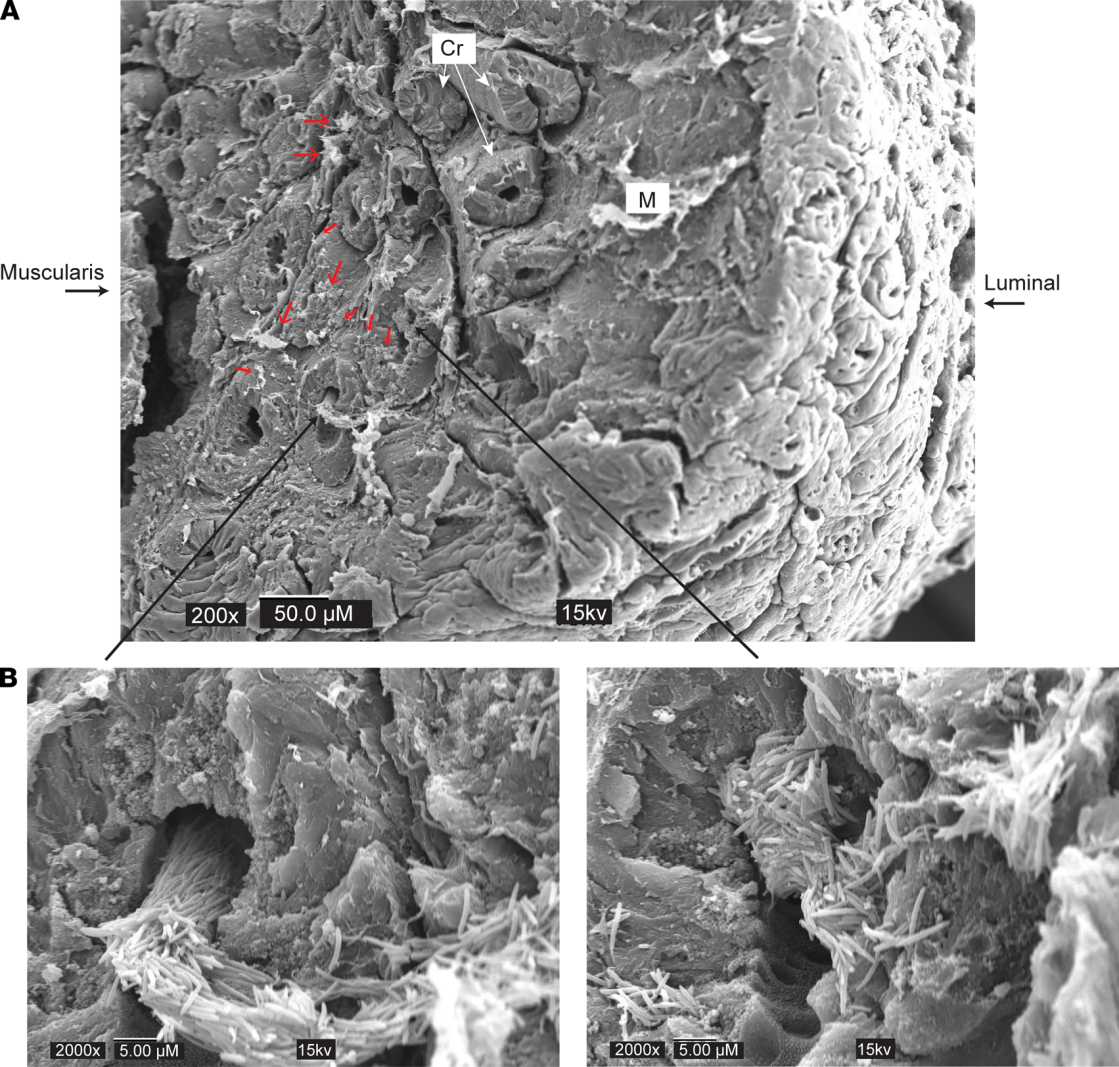
Scanning electron microscopy of *STm^ΔaroA^*-treated tumors. Mice bearing CAC colon tumors were given *STm^ΔaroA^* or control vehicle by oral gavage and tissues were taken 24 hours later. Whole sections of colon with tumors were prepared for SEM by glutaraldehyde fixation, dehydration, and freeze drying. Tumors were cut on the sagittal plane and mounted for platinum coating and SEM imaging. (**A**) Top image shows lower magnification view of a tumor area. Scale bar: 50 μm. Luminal side indicates the top of the tumor that was facing the intestinal lumen, and muscularis side indicates the inner side of tumor reaching the lamina propria and muscularis mucosa. Small red arrows indicate small *STm^ΔaroA^* colonies or individual bacteria. (**B**) Large black arrows indicate areas shown in higher magnification. Scale bar: 5 μm. Cr, Crypt; M, Mucous.

We next employed scanning electron microscopy (SEM) to view bacterial colonization in greater detail. Colonic tumors were analyzed 24 hours after administration, which showed the greatest colonization of *STmΔaroA*. Exceptionally large colonies of *STmΔaroA* were found within the tumor mass just 24 hours after administration (Figure 2, see insets). These were reminiscent of previous observations by Crull et al., in which they found large extracellular colonies of *S*Tm in CT26 tumors 2 days after administration (25). The large size of the bundles suggested that they were rapidly dividing within the tumor extracellular spaces. This is consistent with the CFUs observed at this time point (Supplemental Figure 1) and suggests that initial seeding of the tumor results in a dramatic proliferation of the bacteria, which then recedes. We could also find instances of single or multiple bacteria (Figure 2, red arrows). No bacteria could be observed in nontreated mice (Supplemental Figure 3, A–D), strongly implying that normal microbiota are not penetrating tumor tissue to form mass colonies as observed with the *STmΔaroA*. It is likely that small amounts of microbiota do invade via the disrupted barrier as previously described (26); however, this would be difficult to detect with SEM. IF staining detecting mCherry-expressing *STmΔaroA* further supports the SEM data showing large aggregates of *STmΔaroA* commonly occurring, with some punctate staining indicating individual bacterium (Supplemental Figure 4). Supplemental Figure 5 shows the histological appearance of colon after CAC induction in nontreated and *STmΔaroA*-treated mice, with boxes indicating the type of region imaged in the IF staining of *STmΔaroA* in Supplemental Figure 4.

### STm^ΔaroA^ treatment does not alter the colonic microbiota.

Infection with WT *S*Tm induces changes in the microbiota, which lead to and support an inflammatory environment within the intestine that favors *Salmonella* growth (27). In addition, different microbiomes have been associated with better outcome in cancer and cancer therapy with checkpoint blockades (28, 29). We therefore assessed whether oral administration of *STmΔaroA* altered the microbiota composition. Colonic content was taken from AOM/DSS-induced mice following 6 weeks of treatment with *STmΔaroA* (as per Figure 1A) and subjected to 16s rRNA-Seq. The observed total number of operational taxonomic units (OTUs) was not different between nontreated and *STmΔaroA*-treated mice (Supplemental Figure 6A). Analysis of the α diversity using multiple statistical models (Chao1 and the Shannon and Simpson’s Diversity Index) also showed that there were no differences between the abundance or evenness of microbial species present in nontreated and *STmΔaroA*-treated mice (Supplemental Figure 6B). Analysis by weighted UniFrac for β-diversity also showed no differences in the quantitative abundance of species between groups (analysis of similarities [ANOSIM] test; *r* = 0.214, *P* = 0.068) (Supplemental Figure 6C). This would suggest that, unlike infection with WT *Salmonella*, *STmΔaroA* infection does not elicit changes in the microbiome at the time point tested, which would be consistent with the very low levels of infection in normal tissue (Supplemental Figure 1). There remains the possibility that the microbiota is altered during initial exposure to *STmΔaroA* when its abundance in the gut lumen is higher. However, Supplemental Figure 1 shows that *STmΔaroA* is rapidly cleared from the feces.

To further test whether the microbiota is involved in the efficacy of BCT, we induced colorectal tumors in germ-free (GF) mice using AOM and DSS and then treated by oral gavage with *STmΔaroA*. GF mice are incredibly sensitive to DSS treatment due to reduced barrier function and altered mucosal immunity (30); therefore, even with low dose DSS, weight loss was extreme and many mice reached the ethical end point. The remaining GF mice (4) were treated either with PBS or *STmΔaroA* (1 × 10^7^ CFU) by oral gavage. GF mice showed susceptibility to the attenuated *STmΔaroA* strain and displayed rapid weight loss, which was then maintained (Supplemental Figure 6D). Mice therefore only received 1 dose of *STmΔaroA* and were sacrificed 11 days after the treatment, and tumor burden was analyzed. Given the caveat of there being 2 mice per group, there was a clear abolition of tumors in the *STmΔaroA*-treated GF mice (Supplemental Figure 6E). These mice did have areas of hyperplasia, which were increased compared with NT mice and may represent the former tumor areas (Supplemental Figure 6E). Because mice showed signs of systemic infection (weight loss), we checked the CFU in the spleens and indeed found dissemination of *STmΔaroA* (Supplemental Figure 6F). These data show that the presence of microbiota may, to a degree, impede *STmΔaroA* persistence, likely through competition for space within the intestine. However, GF mice are susceptible to bacterial dissemination, demonstrating the necessity of the microbiota to instruct barrier function. Altogether, these data imply that the presence of the gut microbiota can control the outgrowth of *STmΔaroA*, but there are no appreciable alterations in the gut microbiota that might explain the treatment outcome.

### STm^ΔaroA^ alters the transcriptional landscape of tumors.

**Figure 3:**
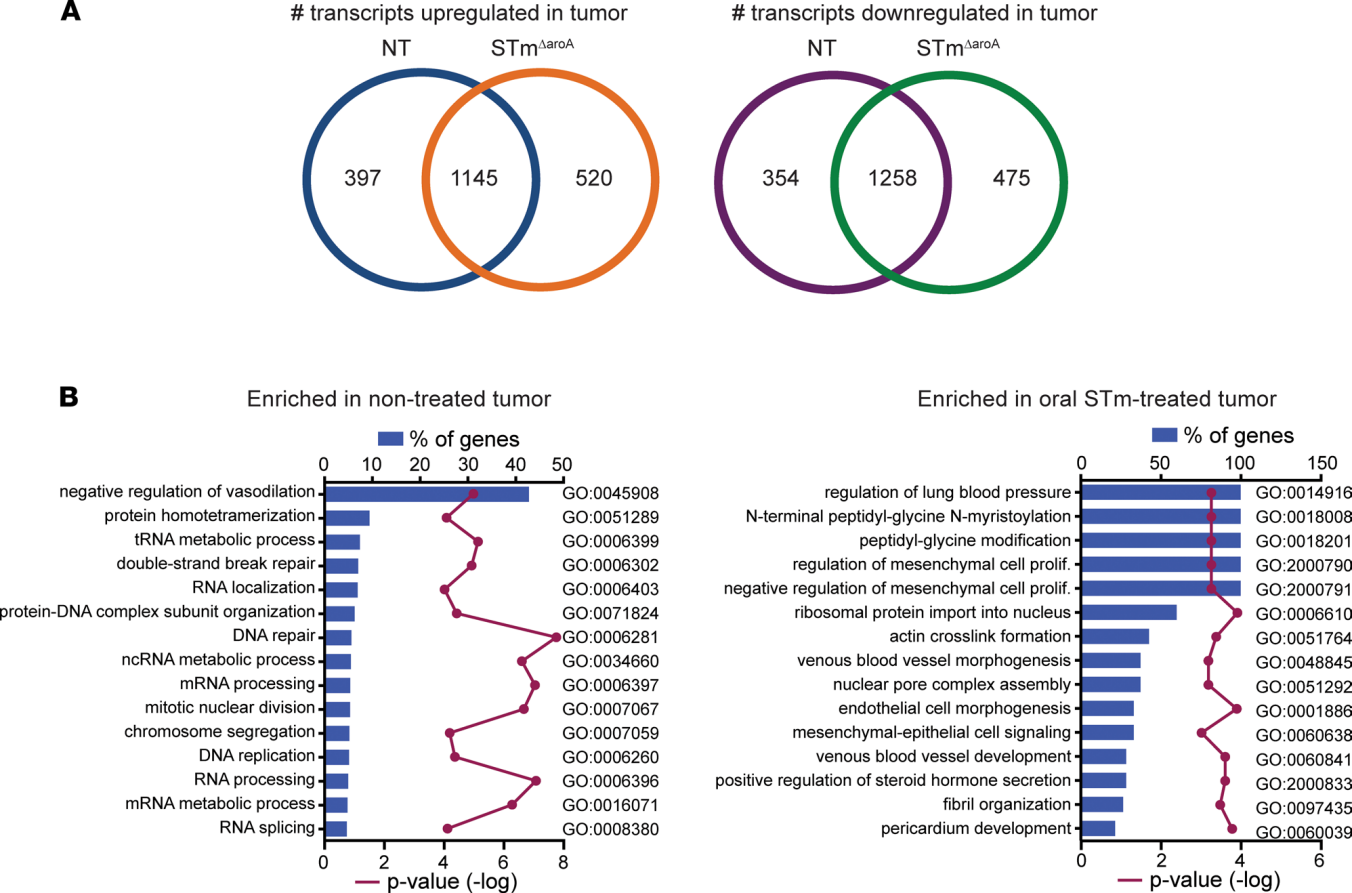
*STm^ΔaroA^* treatment alters the transcriptional landscape of tumors. Normal and tumor tissue were dissected from CAC-bearing mice after 4 weeks of *STm^ΔaroA^* or control treatment. RNA was isolated and used for RNA-Seq analysis. (**A**) Number of transcripts upregulated or downregulated in tumor compared with normal tissue, with overlapping and unique transcripts depicted. (**B**) Differentially expressed genes (DEGs) in tumors compared with normal tissue for each treatment identified in **A** were compared by GO analysis. Data represents the percentage of genes of a given pathway that are enriched in either nontreated or treated tumors, with –log *P* value.

Next, to gain an understanding of the differences between nontreated and *STmΔaroA*-treated tumors, we performed RNA-Seq on RNA isolated from whole tumor (T) or adjacent normal tissue (N) dissected from AOM/DSS-induced CAC-bearing mice after 4 weeks treatment. Tumor burden and size for this cohort of mice are shown in Supplemental Figure 7A. Mice treated for 4 weeks with *STmΔaroA* had a trend toward significantly reduced tumor burden and size. Tumors used for RNA isolation was similar between groups (Supplemental Figure 7A). First, we identified the transcripts that were differentially regulated between N and T tissue in the nontreated and *STmΔaroA*-treated groups. Figure 3A shows the number of overlapping and unique genes for each treatment. It is interesting to note that approximately one quarter of genes either up- or downregulated in *STmΔaroA*-treated tumor tissue are unique to *S*Tm treatment. These differentially expressed genes (DEGs) were then analyzed by gene ontology (GO) analysis using DAVID (31, 32), revealing terms enriched in either the nontreated tumors or in the treated tumors, which intriguingly were vastly different (Figure 3B). As expected, nontreated tumors exhibited enrichment of mRNAs involved in cell cycle processes, mitosis, cell division, DNA repair, and more, whereas *STmΔaroA*-treated tumors displayed enrichment of mRNAs for processes involving regulation of mesenchymal cell proliferation and mesenchymal-epithelial cell signaling, as well as regulation of blood vessel development (Figure 3B and Supplemental Figure 8). Several genes involved in DNA repair, DNA damage response, RNA synthesis, and epithelial-mesenchymal transition were significantly reduced following *STmΔaroA* treatment (Supplemental Figure 8), suggesting major changes in cell proliferation rates.

There was no signature of inflammatory processes picked up in the RNA-Seq by GO analysis. We checked classically proinflammatory cytokines by transcript and found an increase in *Il1b* mRNA, a trend toward increased *Il6*, but no differences in *Il17*, *Tnfa,*and *Ifny* mRNAs (Supplemental Figure 9A). Analysis of tissue homogenates by Luminex cytokine array found increased levels of IL-1β and TNF-α in tumor tissue compared with normal tissue, but no differences were found between treated and nontreated groups (Supplemental Figure 9B). Other cytokines on the array (including IFN-γ, IL-2, and IL-10) were not detected. This is consistent with another report showing that an auxotrophic *S*Tm mutant does not induce inflammation in the mucosa but still induces protective immunity with mucosal invasion–associated virulence factors driving immunogenicity (33).

**Figure 4:**
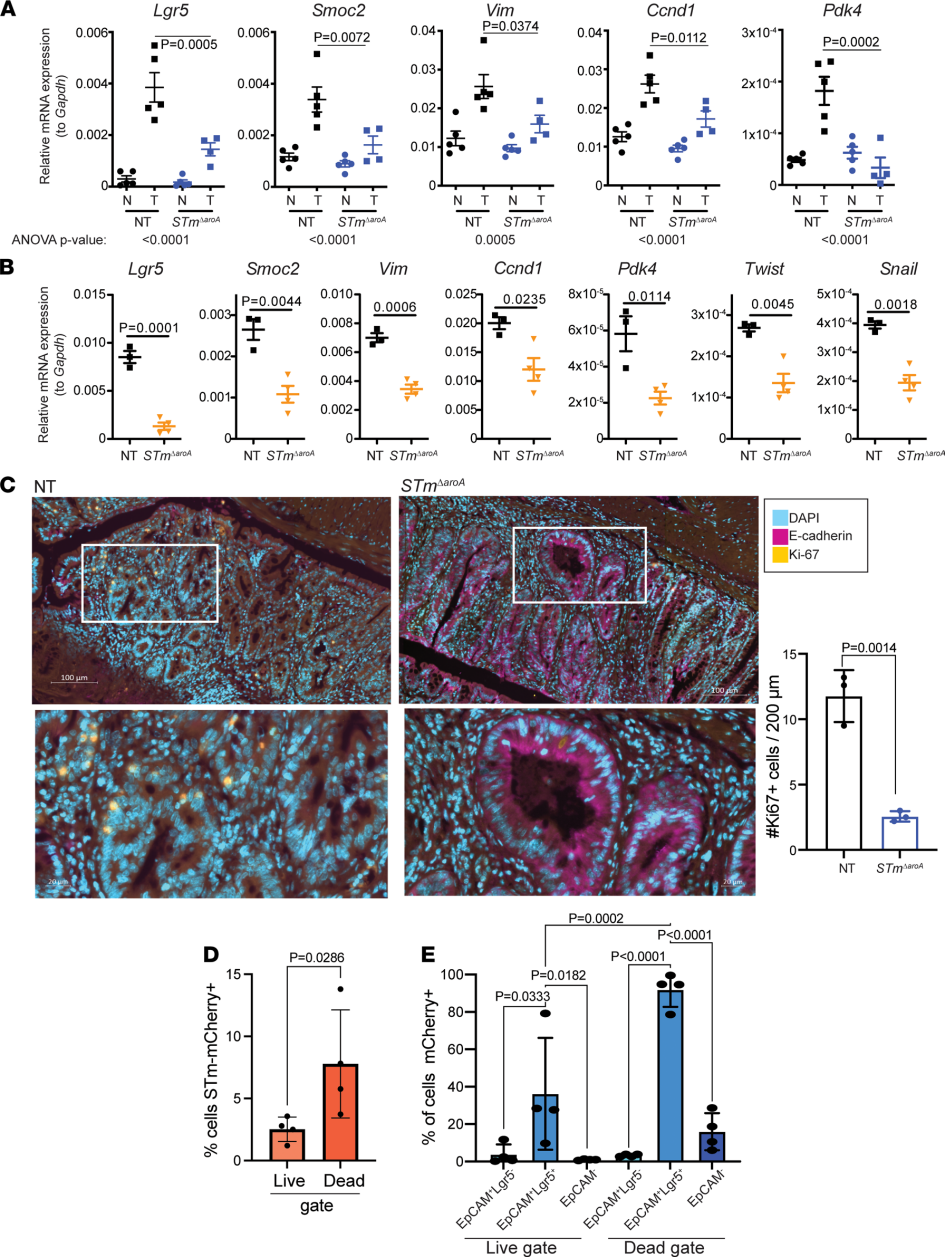
Altered tumor phenotype in *STm^ΔaroA^*-treated mice. (**A**) Quantitative PCR confirmation of genes identified (or pathway related) by RNA-Seq in CAC tumor–bearing mice after 6 weeks of treatment. Nontreated, NT; *Salmonella* treated, *STm^ΔaroA^*; normal tissue, N; tumor tissue, T. Size of tumors used to isolate RNA are shown in Supplemental Figure 7. Data are representative of 3 independent experiments. One-way ANOVA with Turkey’s multiple-comparison test was conducted. ANOVA *P* values are indicated below the graphs, and an individual post hoc test comparing T from each treatment is shown on the graphs. (**B**) Analysis of indicated transcripts in *Apc^min/+^* tumor tissue after 10 weeks of treatment. Data come from 3 (NT) or 4 (*S*Tm) mice shown in Figure 1E. Similarly sized polyps were obtained from each group. Data are representative of 2 independent experiments. Unpaired 2-tailed *t* tests were used. (**C**) Representative immunofluorescence of E-cadherin (purple) and Ki67 (yellow) counterstained with DAPI (blue) in NT and *STm^ΔaroA^*-treated (6 weeks) CAC mice. Scale bar: 100 μm. Lower images are magnification of upper images. Scale bar: 20 μm. For orientation reference, Supplemental Figure 5 shows the type of area (not taken from exact mouse/tumor) imaged here. Quantification of the number of Ki67^+^ cells within 200 μm field of view (FOV) shown to the right. Ten FOV from 2–3 tumors per mouse. Each dot represents the average number for each mouse. (**D**) Lgr5-GFP reporter mice were induced with CAC, as per Figure 1A. Mice were then gavaged with mCherry-expressing *STm^ΔaroA^*, and tumors were collected for flow cytometry analysis 24 hours later. Cells were stained for live/dead marker and EpCAM (CD326); Lgr5-GFP and mCherry were expressed via reporters. Two-tailed Student’s *t* test. (**E**) From **D**, cells were first gated based on EpCAM and Lgr5 expression (as indicated) and the percentage of mCherry^+^ in each population is shown. One-way ANOVA with multiple-comparison post hoc test. Each point represents pooled tumors from 1 mouse. All data are shown as mean ± SD.

Next, we homed in on stem cell, EMT, and metabolism-related genes, and we confirmed a selection of targets by quantitative PCR (qPCR) in independent experiments where mice were treated for 6 weeks. As previously reported, transcripts for epithelial stem cells, proliferation, or epithelial-to-mesenchymal transition–related processes — including *Lgr5* (leucine-rich repeat-containing G-protein coupled receptor), *Smoc2* (SPARC-related modular calcium binding 2), *Vim* (Vimentin), *Ccnd1* (Cyclin D1), and *Pdk4* (pyruvate dehydrogenase kinase 4) (34–40) — were increased in tumor tissue when compared with normal tissue (Figure 4A). Strikingly, these transcripts were largely decreased following *STmΔaroA* treatment (Figure 4A). We confirmed these mRNA changes in the *Apc^min/+^* model, comparing tumor tissue from nontreated and *STmΔaroA* treatment. In line with results from the CAC model, *STmΔaroA* treatment altered the transcriptional levels of the above-mentioned genes and additional EMT-related genes *Twist* and *Snail* (Figure 4B). We also analyzed gene expression in normal, tumor (control-treated) or hyperplasia (*STmΔaroA*-treated) colon tissue from GF mice (from Supplemental Figure 8B) by qPCR. Tumors from GF mice showed similar upregulation of stem cell–associated, mesenchymal, proliferation, and metabolic genes as observed in specific pathogen–free (SPF) tumor-bearing mice, and the hyperplasic tissue taken from the *STmΔaroA*-treated GF mice looked more similar to normal tissue than to tumors from nontreated GF mice (Supplemental Figure 8B).

Loss of E-cadherin protein expression is an important feature of epithelial-derived tumor progression. *Cdh* (encoding E-cadherin) was consistently decreased at the mRNA level in tumors and showed a trend toward increasing in *STmΔaroA*-treated tumors (not significant in all experiments; data not shown). Since translation and protein localization of E-cadherin is important for its function (41), we checked E-cadherin protein expression by IHC staining of sections taken from CAC tumor–bearing mice. Nontreated tumor sections showed very little E-cadherin protein (Figure 4C). In contrast, tumors from *STmΔaroA*-treated mice showed significantly higher levels of E-cadherin within tumor areas (Figure 4C). Thus, it appears that *STmΔaroA* treatment diminishes tumors, reducing tumor stemness markers and restoring epithelial identity. As we had observed enrichment of proliferation-related genes in NT tumors compared with tumors from *S*Tm-treated mice, and decreased tumor size, we assessed proliferation within tumors by Ki67 staining at 6 weeks after treatment. There was an increase in Ki67^+^ cells in NT tumors compared with *STmΔaroA*-treated tumor sections (Figure 4C), which is consistent with the transcriptomic and macroscopic changes in the tumor size. This likely reflects the change in tumor size at this time point. *STmΔaroA* infection of tumor cells was assessed by flow cytometry, and we found that mCherry^+^ cells were more likely to be within the dead gate (Figure 4D). This aligns with previous findings in the literature that intracellular infection can lead to cell death by a range of cell-death pathways (12, 42–44) and implies discreet induction of cell death where bacteria do invade intracellularly. More intriguingly, by use of an Lgr5-GFP reporter mouse, we observed that a higher proportion of Lgr5^+^ cells was infected compared with other epithelial (EpCAM^+^Lgr 5^–^) or nonepithelial cells, with up to 90% of Lgr5^+^ cells being mCherry^+^ in the dead gate and around 30% in the live gate, compared with other cell types, which showed around 1%–5% of cells infected (Figure 4E). It has previously been reported that *Salmonella* and other intracellular pathogens preferentially invade mitotic and dividing cells; thus, Lgr5^+^ stem cells may be more prone to infection (45, 46). This raises the interesting possibility that *S*Tm could be used as a tool to directly affect cancer stem cells. However, it is unlikely that *S*Tm will reach every tumor stem cell necessary (see *S*Tm form distinct extracellular colonies) to eradicate the tumor by that mechanism alone, and many other cells types play a role in tumor progression. Taken together, these data show that *S*Tm can potently modulate the transcriptional landscape of tumors, and reduction in stem cell–associated transcripts is supported by the flow cytometry analysis showing an accumulated of infected Lgr5^+^ cells within the dead fraction.

### STm^ΔaroA^ alters the metabolic environment of tumors.

**Figure 5:**
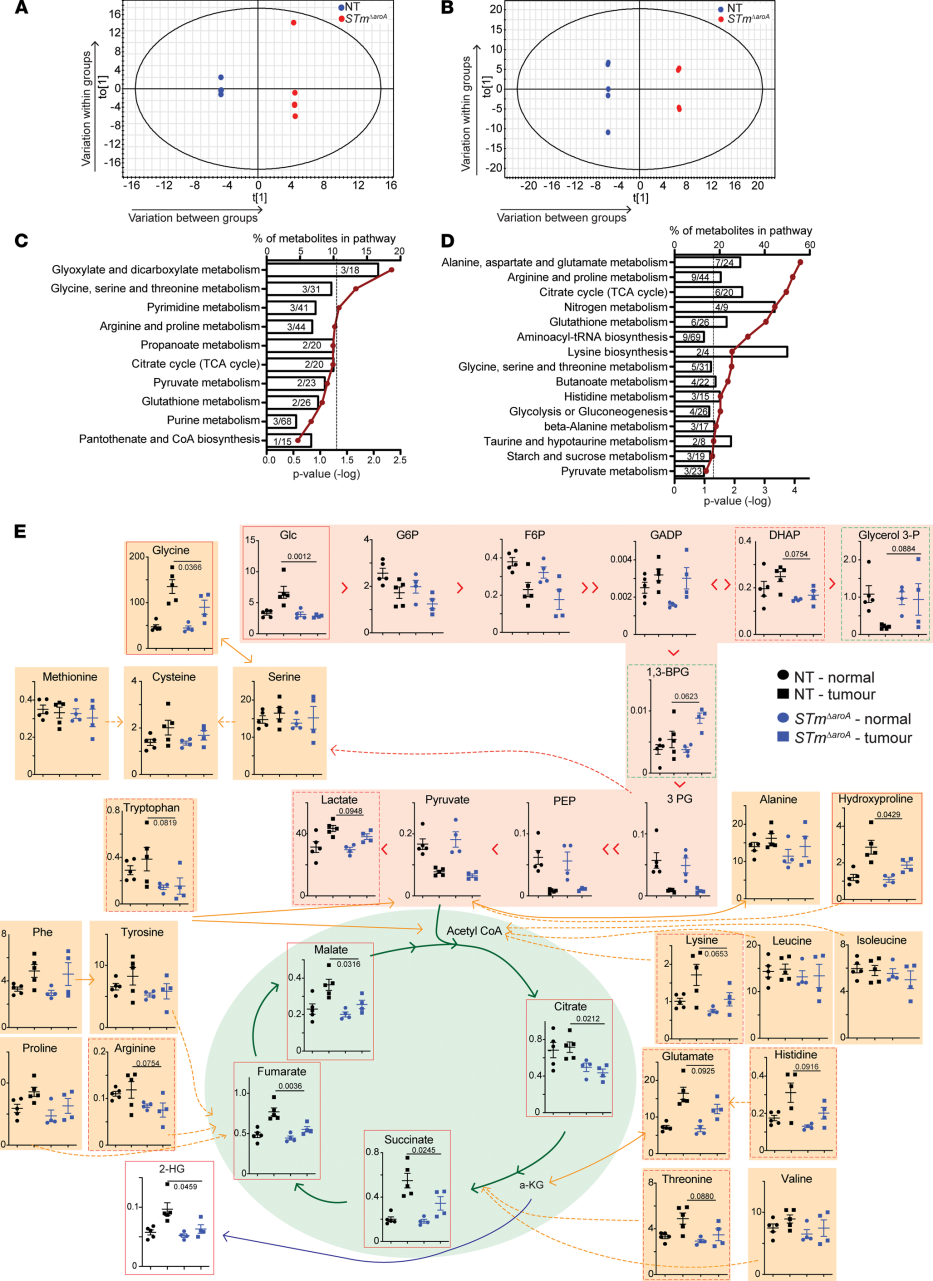
*STm^ΔaroA^* treatment alters the metabolic environment of CAC tumors. Tumor metabolites of CAC-induced colon tumors were assessed by GC-MS. (**A** and **B**) OPLS analysis of metabolites comparing nontreated (NT) and *STm^ΔaroA^*-treated tumors after 6 weeks (**A**) and 24 hours (**B**) of treatment. The size of tumors used for analysis is shown in Supplemental Figure 7, B and C. All metabolites significantly different between *STm^ΔaroA^*-treated and nontreated tumors (VIP score > 1) were submitted to pathway analysis (MetaboAnalyst). (**C** and **D**) Pathway analysis for 6 weeks of *STm^ΔaroA^* treatment (**C**) and 24 hours treatment (**D**), represented as the percentage of metabolites in a pathway that were altered, against *P* value (–log); hypergeometric test used. (**E**) Metabolites detected from glycolysis (pink shading) and TCA cycle (green shading), and amino acids (orange shading), with interrelationships depicted (24 hours after treatment). The *x* axis shows nmol/g. One-way ANOVA was performed with Bonferroni multiple-comparison test; *P* values shown are the multiple-comparison statistic. Data are shown as mean ± SD. Both 6-week and 24-hour analyses were performed on 2 independent experiments, with similar changes observed in both sets.

Previous studies have demonstrated that BCT can affect tumor growth by utilizing excess nutrients, such as ethanolamine (47), or are attracted to tumors due to high levels of metabolites such as ribose or leucine (48). Our observation of large intratumoral, extracellular *STmΔaroA* colonies led us to question whether the tumor metabolome would be altered following treatment. From 4 hours to 24 hours after infection, there is a large increase in CFUs, and along with the appearance of the microbes in the SEM analysis (Figure 2) we hypothesized that bacteria would be rapidly dividing and, therefore, competing for essential metabolites within the tumor environment. Tumor and normal tissue from nontreated or *STmΔaroA*-treated CAC–tumor-bearing mice after 6 weeks or 24 hour of treatment were analyzed by gas chromatography–mass spectrometry (GC-MS) analysis for polar metabolites. Unit variance–scaled (UV-scaled) GC-MS data were analyzed, and orthogonal partial least squares–discriminant analysis (OPLS-DA) plots revealed a separation between nontreated and treated tumors after 6 weeks — but more importantly within 24 hours (Figure 5, A and B) (6 weeks treatment in vivo, *R*^2^ = 0.99; *Q*^2^ = 0.52; 24 hours treatment in vivo, *R*^2^ = 0.99; *Q*^2^ = 0.67). It is possible that the alteration in the metabolome status at 6 weeks could be due to the reduced tumor burden. However, we selected remaining larger tumors for analysis, which did not differ overall in size (Supplemental Figure 7B), though this does not rule out altered tumor characteristics. Importantly, at 24 hours, there is no difference in tumor burden between treatment groups (Supplemental Figure 7C); however, we observed dramatic changes in the metabolome, which is concurrent with the large increase in bacterial CFU, illustrating a direct impact of *STmΔaroA* on the tumor metabolic environment early after invasion. This precedes reduction in tumor size and likely aids in driving the reduction in tumor burden.

We performed pathway analysis on metabolites with a variable importance on the projection (VIP) score greater than 1 using MetaboAnalyst 3.0 (49, 50) (Supplemental Tables 3 and 4 show the complete list). Common pathways affected by *STmΔaroA* treatment at both time points (6 weeks and 24 hours) included glycine, serine, and threonine metabolism; arginine and proline metabolism; and citric acid cycle, among others (Figure 5, C and D). As previously described (19), many metabolites — and particularly amino acids and tricarboxylic acid (TCA) cycle intermediates — are increased in tumor tissue compared with normal tissue (Figure 5E and Supplemental Figures 10 and 11). This likely reflects the increased energy and anabolic requirements of tumors. Strikingly, many metabolites were decreased following *STmΔaroA* treatment. Figure 5E shows metabolites detected in glycolysis and the TCA cycle, as well as amino acids 24 hours after treatment. Perhaps not surprisingly, glucose was significantly reduced in *STmΔaroA-*treated tumors (Figure 5E). Other glycolysis intermediates were only mildly affected and glycerol-3-phosphate, and 1,3 bisphosphoglycerate (1,3-BPG) trended to increase in *STmΔaroA-*treated tumors. Several TCA cycle intermediates (citrate, succinate, fumarate, and malate) were reduced following treatment (Figure 5E). Furthermore, several amino acids, which can feed different parts of the TCA cycle were reduced. This included glutamate (glutamine was not detected) (Figure 5E), which is an important fuel for glutaminolysis, upon which tumors can be quite dependent (51).

Other important oncometabolites were also affected. The polyamine synthesis pathway appears affected at both 24 hours and 6 weeks after treatment. Ornithine was reduced in *STmΔaroA-*treated tumors 24 hours and 6 weeks after treatment, while putrescine was significantly affected after 6 weeks of treatment and spermidine after 24 hours (Supplemental Figures 10 and 11). 2-Hydroxyglutarate (2-HG) can accumulate in tumors due to mutant or overactive isocitrate dehydrogenase 1/2 (IDH2) activity, converting α-ketoglutarate (α-KG) to 2-HG, which can inhibit α-KG–dependent dioxygenases, leading to increased histone and DNA methylation (51). We saw increased 2-HG in CAC tumors, and this was decreased after 24 hours of treatment (Figure 5E). Thus, several important fuel sources, metabolic intermediates, and oncometabolites are decreased following *STmΔaroA* treatment, presumably through metabolic competition between the bacteria and tumor cells.

### STm^ΔaroA^ directly affects tumor epithelium.

**Figure 6:**
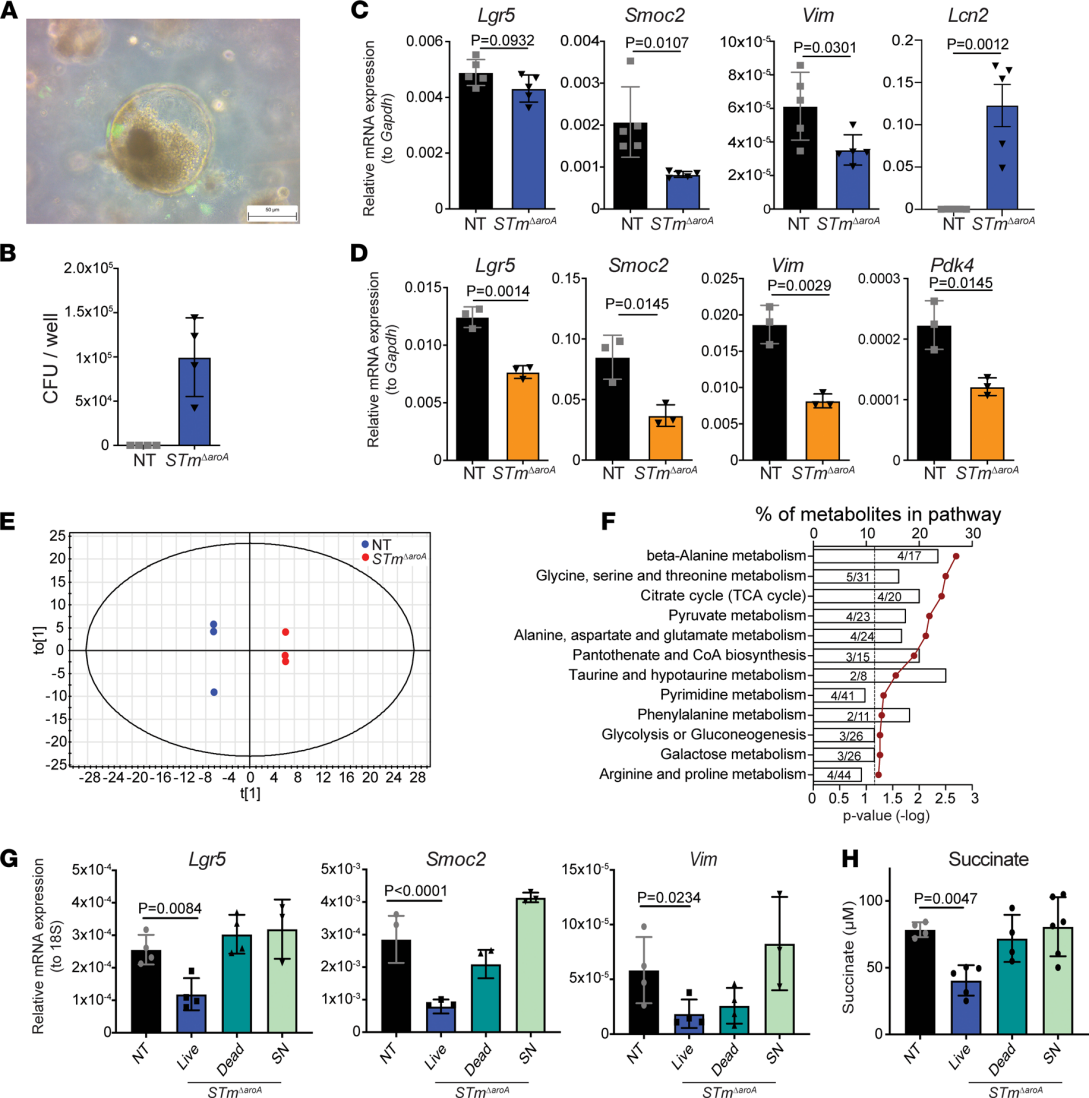
In vitro treatment of tumor-derived organoids with *STm^ΔaroA^*. Tumor organoids derived from CAC-induced tumors and *Apc^min/+^* tumors were established and infected with GFP-expressing *STm^ΔaroA^* for 2 hours. Infection was washed off, and then, organoids were cultured with gentamycin for a further 24 hours. (**A**) Merged bright-field and fluorescence microscope image of organoids within matrigel after 24 hours of infection shows association of *STm^ΔaroA^* with tumor organoids. Scale bar: 50 μm. (**B**) CFU of *STm^ΔaroA^* per well after 24 hours of infection. (**C** and **D**) qPCR analysis of the indicated transcripts in CAC-derived (**C**) and *Apc^min/+^* tumor (**D**) organoids. Representative of > 3 independent experiments with 4 independently derived organoid lines, with between 3 and 5 technical replicates per experiment. One replicate is 1 well of a 24-well plate culture with a 50 μL Matrigel dome. (**E** and **F**) Tumor organoid metabolites were assessed by GC-MS, OPLS-DA analysis, and pathway analysis of metabolites with a VIP score > 1. (**G**) CAC-derived tumor organoids were cultured with live or heat-killed (dead) *STm^ΔaroA^* or with supernatant (SN) of *STm^ΔaroA^* grown in organoid culture medium and the indicated mRNAs analyzed by qPCR. (**H**) Analysis of succinate levels in tumor organoids treated as described in **G**. Individual 2-tailed Student’s *t* tests (**C** and **D**) or Kruskal-Wallis tests (**G** and **H**) were performed. Data are shown as mean ± SD.

Our initial hypothesis was that BCT would have a direct effect on tumor epithelium. The effects that we have described so far on tumor stem cell markers, namely an increase in epithelial identity and a change in the tumor metabolome in *STmΔaroA*-treated tumors, suggests an impact of *STmΔaroA* treatment on the tumor environment and on tumor cells. To directly test this hypothesis, we utilized tumor 3D organoid cultures. We generated tumor organoid lines from CAC-induced colorectal tumors and from *Apc^min/+^* SI and colonic polyps/tumors. These grow independently of exogenous Wnt pathway agonists (R-spondin and noggin), and the only supplement provided in the culture was EGF (52). Representative images of organoid appearance are shown in Supplemental Figure 12, A–C. Tumor organoids were infected with *STmΔaroA* by inoculating the culture medium (1 × 10^8^ CFU). *STmΔaroA* were able to invade the Matrigel and infect the organoids (Figure 6A and Supplemental Figure 12B). After 2 hours of infection, the culture medium was washed off, and fresh medium containing gentamycin was added, so only bacteria that had infected organoids could grow, preventing any effects purely from bacterial overgrowth. Organoids were then collected for analysis 24 hours after the initial infection. CFU analysis was performed to determine bacterial burden (Figure 6B). We detected approximately 1 × 10^5^ CFU per well (Supplemental Figure 12D), which contains around 1 × 10^6^ cells within the organoid structures. Importantly, treatment of organoids with *STmΔaroA* could recapitulate effects on gene expression seen in vivo, with a substantial reduction in transcripts for *Lgr5*, *Smoc2*, and *Vim* in both CAC-derived and *Apc^min/+^*-derived tumor organoids, as well as *Pdk4* in *Apc^min/+^* organoids (expression was very low in CAC organoids) (Figure 6, C and D). As seen with the RNA-Seq data set (Figure 3), transcripts were not only decreasing after *STmΔaroA* treatment, but they showed dynamic changes. For example, an innate immune protein known to respond to bacterial infection, lipocalin-2 (*Lcn2*) (53), shows robust induction following organoid infection (Figure 6C). This confirms that the reduction in specific transcripts — for example, affecting stem markers — is not a global transcriptional repression. Of note, mRNA quality and amount was consistently similar between treatment groups, and Ct values for housekeeping genes were also the same between groups, showing that decreases in certain transcripts are not due to dying cells

Next, we tested whether *STmΔaroA* treatment in vitro would have an effect on the cellular metabolome of the organoids. As with the in vivo findings, the organoid metabolome demonstrated separation of nontreated and treated organoids by OPLS analysis (Figure 6E). Taking all metabolites with a VIP score > 1 (Supplemental Table 5) and analyzing by MetaboAnalyst revealed similarly affected metabolic pathways following in vitro *STmΔaroA* treatment as for in vivo treatment, with amino acid metabolism pathways, TCA cycle, and glycolysis being altered (Figure 6F and Supplemental Figure 13). These data suggest that bacterial colonization imposes direct metabolic competition, leading to an altered cellular metabolome. These results provide evidence that *STmΔaroA* treatment can directly affect the tumor cells, independently of effects involving other systems/cell types, such as the immune system.

To further dissect whether live bacteria are required to mediate the observed effects of *STmΔaroA* on tumor organoids, or whether the presence of heat-killed bacteria or bacterial supernatant (SN) would be sufficient, we compared treatment of tumor organoids with live *STmΔaroA*, heat-killed *STmΔaroA*, or *STmΔaroA* SN (prepared using 10 kDa exclusion columns). Live bacteria had the strongest effect on reducing stem cell and EMT marker expression (Figure 6G). Heat-killed bacteria induced a slight reduction in *Smoc2* and *Vim*, while *STmΔaroA* SN had no effect (Figure 6G), suggesting that secreted products from bacteria are not exerting these antitumor effects (note 10 kDa filters exclude LPS; Supplemental Figure 12D). Furthermore, succinate, one of the metabolites identified as being reduced by *STmΔaroA* treatment in vivo, was measured, and only live *STmΔaroA* treatment resulted in reduced levels (Figure 6H), further supporting the idea that live *STmΔaroA* directly impose metabolic competition.

### An effect of STm^ΔaroA^ treatment on tumor organoid stem–forming capacity.

**Figure 7:**
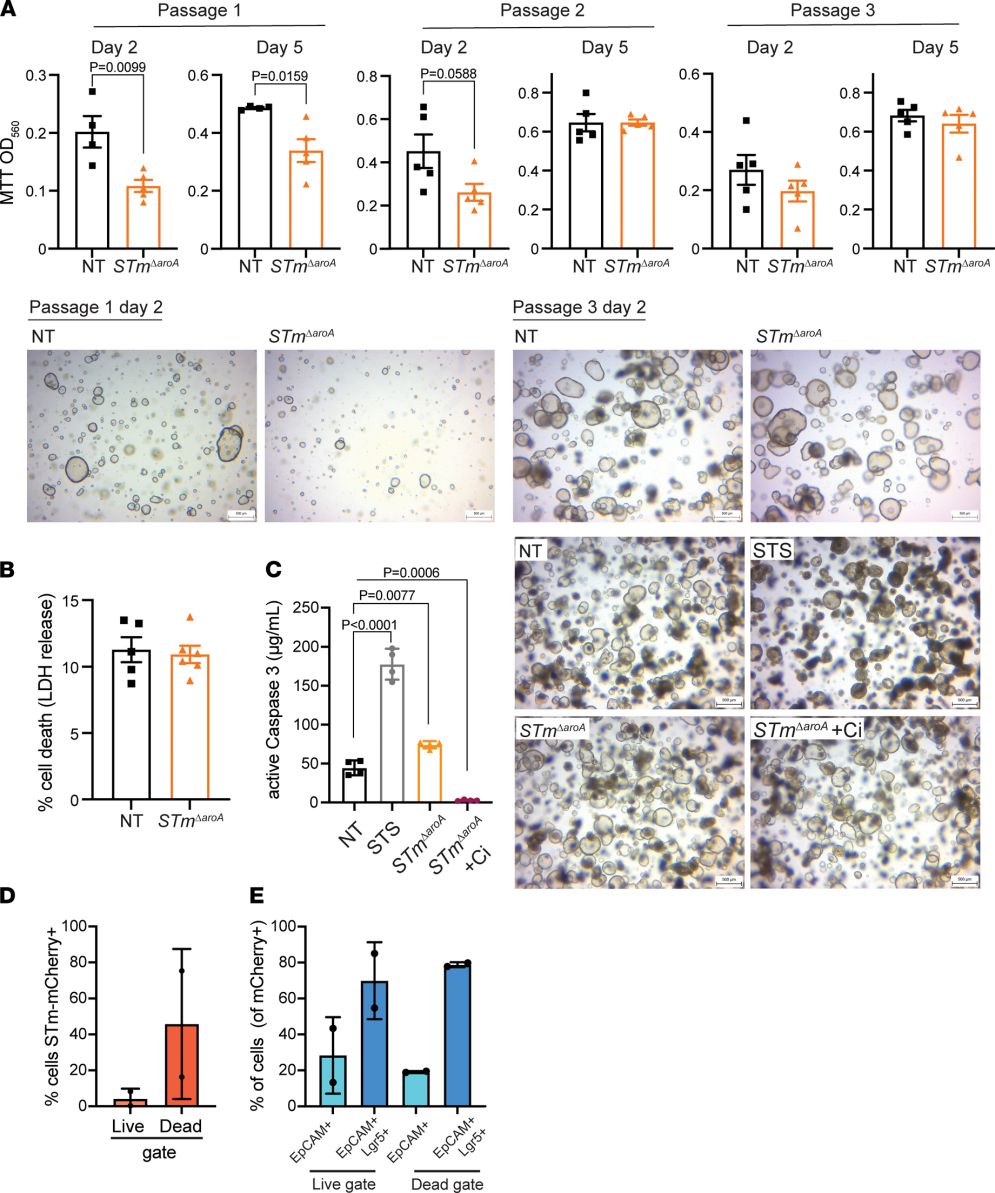
*STm^ΔaroA^* treatment affects tumor organoid stem–forming capacity. (**A**) Organoids were infected with *STm^ΔaroA^* (or control) as in Figure 6 for 24 hours. They were then dissociated into a single cell suspension. An equal number was then reseeded into Matrigel and passaged weekly at an equal density for 3 weeks. MTT assay was performed at the indicated day. Representative images are shown below for the indicated days. Scale bars: 500 μm. Each point indicates an independent well. Two-way Students T-test performed. Representative of 2 experiments, data shown from *Apc^min/+,^*SI tumor line. (**B**) Measurement of LDH in the cell culture supernatant after 24 hours of infection. Data shown as percentage of cell death compared with wells treated with cell lysis solution. Each data point indicates an independent well. Data are representative of 3 experiments. (**C**) Active caspase 3 assessed by a plate-based colorimetric assay on organoids infected as in **B**, with the addition of a pan-caspase inhibitor or staurosporine (STS) alone. Each point is an individual well. One-way ANOVA with Dunnett’s multiple-comparison test. Representative images to the right. Scale bar: 500 μm. Data are representative of 2 independent experiments and shown from *Apc^min/+,^*SI tumor line. (**D** and **E**) Tumor organoids derived from Lgr5-GFP reporter mice induced with CAC were infected with mCherry-expressing *STm^ΔaroA^* for 24 hours, as outlined. Organoids were dissociated into single cells, stained with a live/dead marker, and analyzed by flow cytometry. The percentage of cells that are infected (mCherry^+^) in the live or dead cell gate (**D**) and the percentage of cells from the mCherry^+^ gate that are EpCAM^+^Lgr5^–^ or EpCAM^+^Lgr5^+^ (**E**) are shown. Data are pooled from 2 independent experiments, and each point is an average from 2 wells. Data are shown as mean ± SD.

As we observed effects of stem cell–related transcripts after *STmΔaroA* treatment both in vivo and in vitro, we assessed the effect of *STmΔaroA* treatment on organoid-forming capacity. Colon-derived tumor organoids were either PBS- or *STmΔaroA*-treated for 24 hours (as described in Figure 6) and were then dissociated into single cells. Cell counts were performed, and equal numbers were reseeded and regrowth was followed over subsequent passages. We found that *STmΔaroA*-treated organoids had reduced capacity to regrow in the first 2 passage, with reduced cell number (Supplemental Figure 14, A–C) and growth as measured by MTT assay (Figure 7A). *STmΔaroA*-treated organoids recovered to the same density by late passage 2 into passage 3 (Figure 7A and Supplemental Figure 14, A–C). It is worth noting that *STmΔaroA* did not persist in the organoids when we dissociated them for reseeding. Infection by Salmonella can lead to inflammasome activation, pyroptosis, and release of LDH into the culture medium (12, 42–44). We first tested cell death first by LDH release, and we found no increase in LDH release over 24 hours in *STmΔaroA*-treated organoids compared with NT (Figure 7B). We also assessed active caspase 3, as this may be more sensitive to localized cell death. There was a moderate, yet significant, increase in active caspase 3 levels upon *STmΔaroA* treatment, which was reversible with a pan-caspase inhibitor (Figure 7C). Treatment with staurosporine (STS) showed much more robust induction of caspase 3 (Figure 7C). Thus, while there was a degree of apoptosis occurring with *STmΔaroA* treatment, it was not completely cytotoxic. This is consistent with the estimate that approximately 1:10–1:100 (experimental variability) cells within the organoid culture are infected. Given that a small number of cells are infected by *S*Tm in vivo, and Lgr5^+^ cells showed greater propensity to be infected and undergo cell death, we assessed Lgr5-GFP tumor organoids by flow cytometry. Similar to what we observed in vivo, mCherry^+^ cells were more likely to be within the dead gate (Figure 7D), and of mCherry^+^ cells, there was a higher proportion that were Lgr5^+^ (Figure 7E). Altogether, these data show that *S*Tm treatment resulted in a low level of cell death that appears to predominantly affect the Lgr5^+^ stem compartment.

### A single dose of STm^ΔaroA^ in vivo can reduce tumor burden.

**Figure 8:**
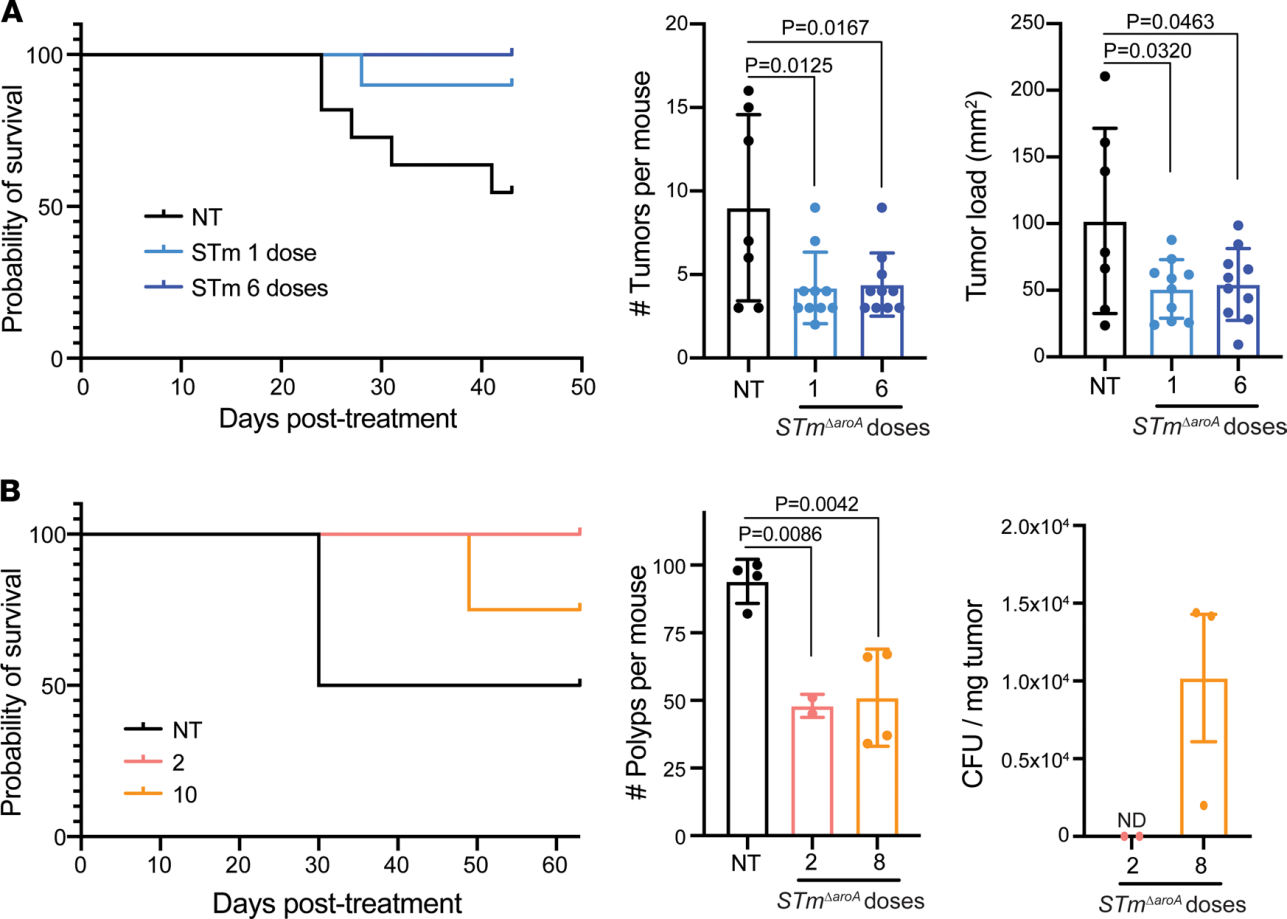
Shorter treatment regimens of *STm^ΔaroA^* yield similar protection. (**A**) AOM/DSS CAC was induced as per Figure 1A in female C57B6/J mice. Mice were then split into no treatment (NT, PBS control) and 1 or 6 doses of *STm^ΔaroA^* (given once per week via oral gavage). The left is survival from treatment start point (*P* = 0.0184 Mantel-Cox log-rank test), the middle is the tumor burden, and the right is tumor load. (**B**) *Apc^min/+^* mice were treated from 9 weeks of age with either PBS control (NT), 2 doses of *STm^ΔaroA^* (with PBS control for remaining 4 weeks), or 6 doses of *STm^ΔaroA^*. The left is survival (Mantel-Cox log-rank test), the middle is number of polyps per mouse (small intestine), and the right shows CFU obtained from polyps at necroscopy. Data are shown as mean ± SD.

Since we see an initial effect of *STmΔaroA* on tumor organoids that subsides once the bacteria are removed, we surmised that the continual dosing of *S*Tm would be required in vivo, since they do decline over time in tumors (Supplemental Figure 1). We treated CAC-induced and *Apc^min/+^* tumor–bearing mice with either 1 (2 for *Apc^min/+^*) dose, or consecutive weekly dosing as previously. Since these experiments were performed in a different animal facility, we found that overall survival of tumor-bearing mice was reduced compared with previous experiments, with CAC-induced mice developing rectal prolapses due to tumor bulk at the rectum and *Apc^min/+^* mice developing anemia (pale paws being an ethical end point). We found increased survival in CAC-induced mice treated with either 1 or 6 consecutive doses of *STmΔaroA* compared with control-treated mice (Figure 8A). Additionally, there was a significant decrease in the tumor burden and tumor size of mice treated with *STmΔaroA*, in both the 1- and 6-dose groups, compared with control treatment (Figure 8A), indicating that a single dose of *STmΔaroA* is sufficient to exert antitumor effects. Similar to CAC-induced tumor–bearing mice, *Apc^min/+^* mice treated 8 doses had a similar reduction in polyp burden as mice given 2 doses (Figure 8B); in this case, mice received 2 doses in the first 2 weeks and then PBS control for the remaining 6 weeks. Control-treated mice also showed a trend toward decreased survival, as seen in the CAC model (Figure 8B); however, this was not statistically significant, likely due to relative underpowering of the groups. We aimed to asses CFU of tumors or polyps from mice given either the short dosing or continuous *STmΔaroA* dosing. It appears that CFUs have mostly contracted in the 2-week dosing compared with 8 weeks of dosing, which would be consistent with the observation that CFUs diminish at 2 weeks after treatment (Figure 8B; 2 doses yielded just 8 CFU in 1 sample and none in the other). However, we cannot completely exclude colonization below the limit of detection, and despite the resolution of the *STmΔaroA* by the end of the treatment protocol, there is still effective reduction in tumor burden. The idea that 1 or 2 doses is sufficient to reduce tumor burden might indicate that initial outgrowth of *S*Tm within tumors and competition for metabolites are key factors in driving tumor regression, as is induction of cell death in infected stem (and other) cells.

## Discussion

**Figure 9:**
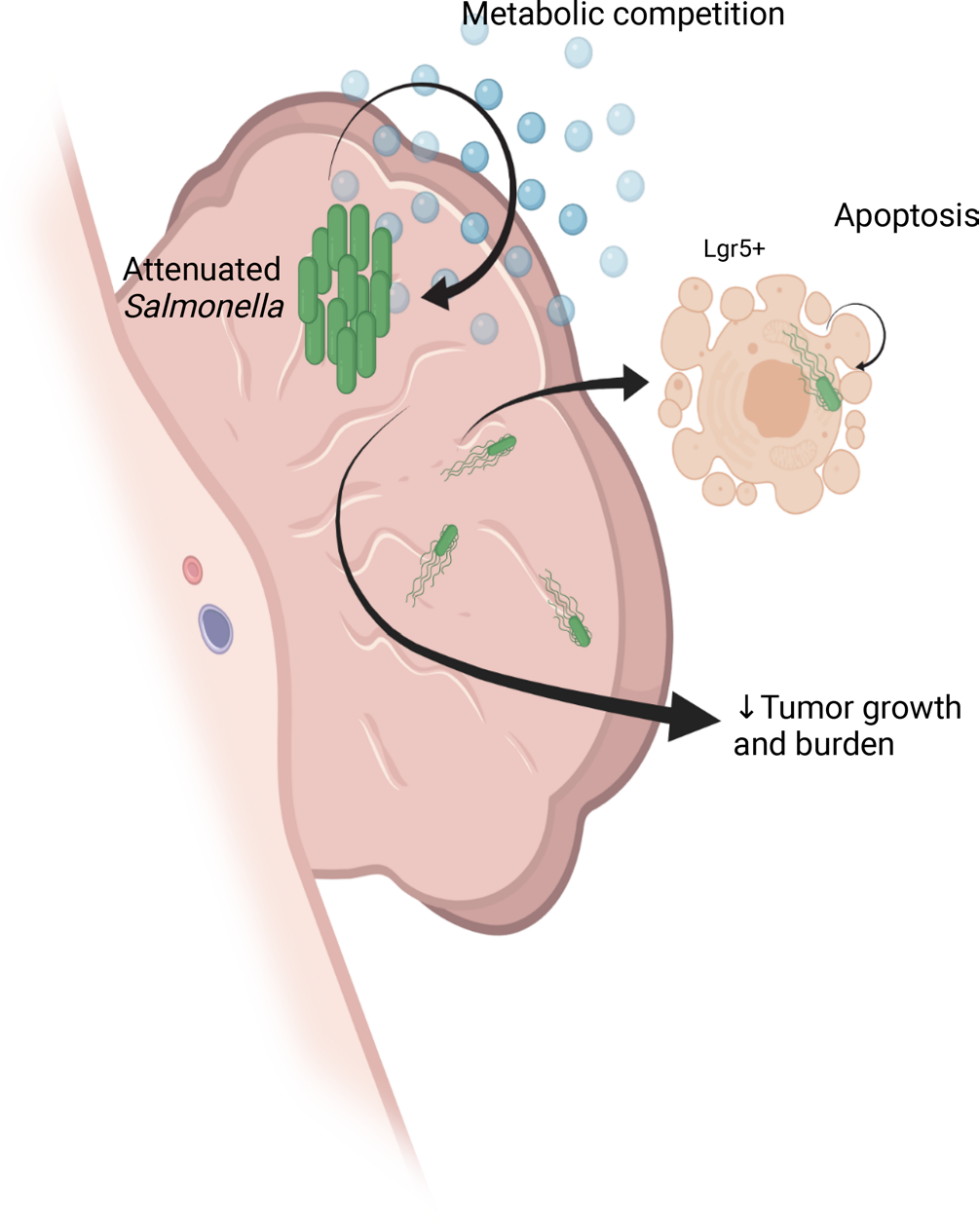
Summary. Attenuated *STm* therapy shows efficacy in mouse models of autochthonous cancers of the intestine. *S*Tm accumulate within 24 hours of oral administration in large intratumoral colonies affecting the tumor metabolic environment. *S*Tm also invade a small proportion of tumor and tumor-associated cells, which undergo cell death. Lgr5^+^ stem cells are preferentially invaded, and accordingly, decreased stem markers can be observed following *S*Tm treatment. In summary, this study highlights the feasibility of oral *S*Tm therapy of colorectal cancer, and it highlights some previously unappreciated effects of bacterial cancer therapy. Figure created with BioRender.com.

In this study, we present data showing that BCT can be efficacious in in situ models of intestinal cancer, and this is the first study to our knowledge to assess oral delivery of BCT in autochthonous CRC models. Oral delivery of *STmΔaroA* to colonic or SI tumor–bearing mice induced a strong reduction in tumor number and size. This was preceded by a dramatic shift in the tumor metabolic landscape, which persisted over treatment. Later, reductions in stem cell–associated, cell cycle, and proliferation-related transcripts were observed, along with a reduction in tumor size. In vitro infection of tumor organoids recapitulated effects seen on the tumor metabolome, and reduced stem cell–associated transcripts were associated with delayed regrowth following withdrawal of *STmΔaroA*. We also observed an overrepresentation of Lgr5^+^ cells that were infected and dying, both in vivo and in vitro, which may explain the reduction in stem cell–associated transcripts observed. This targeting of tumor stem cells, along with metabolic competition, likely drives nonimmune-mediated effects of *STmΔaroA* therapy (Figure 9).

Previous studies have utilized orthotopic or xenograft transplant tumor models, which may not fully recapitulate complex tumor environments in spontaneously formed tumors. Furthermore, studies have delivered bacteria via i.p. or i.v. routes, which, while efficacious in mice, has not been successful in humans. In a phase 1 trial, giving heavily attenuated *S*Tm (VNP20009) i.v. resulted in toxicity and poor tumor localization (16), whereas another small trial administering bacteria by intratumoral injection had better tumor localization (54). Lack of chemotactic ability of the VNP20009 strain, due to mutation of the *cheY* gene, has been suggested to be a limiting factor to its success. Mouse models have shown *cheY* to be redundant (55), while another study has shown it to be important (47), for tumor localization. Crull et al. (14) hypothesized that tumor invasion in vivo is more passive than in vitro, as the resulting chemokine and cytokine release upon i.v. or i.p. delivery of *S*Tm would open tumor vasculature, enabling delivery of bacteria to the tumor. Importantly, the human serum complement system is known to be far more effective than that of mouse (56), and the ΔaroA strain of *S*Tm has been shown to have increased sensitivity to complement due to alterations in the LPS structure (22). Thus, i.v. delivery of *S*Tm in humans likely leads to rapid clearance of bacteria; therefore, more feasible delivery routes need to be considered to move more BCTs into the clinic.

BCG therapy, the only currently approved BCT, is given directly onto the bladder epithelium via intravesicle delivery, where it is thought to directly affect the bladder epithelium via fibronectin interaction, which precedes immune cell recruitment (10). Additionally, Coley’s original experimental treatment involved direct injection into tumors (1). This suggests that BCT may be more effective where it can be applied more locally. Oral delivery of attenuated *S*Tm would feasibly enable targeted colonic tumor delivery while bypassing any i.v. route–associated toxicity. Proof of principle on tolerance and safety of such treatment can be seen with *S*. Typhi vaccination (15).

We tested whether *STmΔaroA* treatment affected the composition of the colonic microbiome and found no significant changes. This is in contrast to infection with WT *Salmonella* (27). One caveat is that we only tested the microbiota at the end point and not early during initial *STmΔaroA* exposure; thus, it is possible that changes could occur earlier during treatment. However, we did not observe any long-lasting effects on microbiome structure. This is encouraging for therapeutic application, since alteration of the microbiome could have unforeseen consequences. In addition, by testing the treatment in GF mice, we found that there were very strong effects when there was no other competition to colonize the gut, as with SPF mice. However, this very artificial system also demonstrates the importance of the microbiota in protecting the host, as even *S*Tm*ΔaroA* could invade and infect systemically. Treating cancer patients with antibiotics prior to surgery is common practice, but in light of the importance of diverse microbiota for controlling cancer (28, 29), it has become apparent that it is not optimal for patient outcome, with antibiotic preconditioning leading to worse outcomes (57–59). Our data do not exclude that there could be certain microbiota compositions that will enable more effective therapy, as with checkpoint blockade therapy (28, 29, 59). Therefore, analyzing microbiomes may be something to consider when starting human clinical trials with orally delivery BCT.

Tumor tissue tropism of attenuated bacteria is thought to be driven by the lack of immune detection within tumors and also the metabolic environment. Previous studies have shown that *S*Tm genes involved in ethanolamine catabolism were advantageous for *S*Tm within tumors (47) and that *S*Tm utilize nutrient-sensing pathways to localize to tumors (48). While the tumor metabolic environment has been suggested to be important for bacterial tumor homing, it was not appreciated what impact BCT might have on the tumor metabolome. We show a dramatic change in the tumor metabolome following *S*Tm treatment. As has been previously reported (19), tumors have higher levels of a wide range of metabolites compared with normal tissue, including sugars; central carbon metabolites; and amino acids, including AAs (Phe, Trp, Tyr) (Figure 4 and Supplemental Figures 7 and 8). We found that *STmΔaroA* form large intratumoral colonies and drastically reshape the tumor metabolome within 24 hours. Multiple metabolic pathways were affected by *STmΔaroA* treatment, which would impose strong metabolic pressure on tumors cells, and this would possibly make it more difficult for tumors to switch from one pathway to another to meet energy and anabolic requirements. Crucially, these biochemical effects were not seen in surrounding normal tissue. Since *STmΔaroA* are auxotrophic for AAs, one might expect greater reductions in AAs than we observed. However, it is clear that the levels of AAs, and many other metabolites, are much more abundant in tumor tissue than in the normal colonic tissue, and so there is likely excess levels required for *STmΔaroA* growth. Targeting tumor metabolism is an important avenue for cancer therapy, with standard chemotherapies taking advantage of metabolic weaknesses (51). However, not all tumors are susceptible, and side effects from inhibiting all fast-dividing cells limit metabolic inhibitor usage. Furthermore, some tumors are able to metabolically adapt if one pathway is blocked (51). BCT may, therefore, be an avenue for introducing metabolic competition; coupling this with other metabolic inhibitors may enable lower doses of these drugs that may otherwise cause severe side effects.

The data presented here show bulk metabolites from tumors that contain *STmΔaroA*, so it is not possible to decipher which metabolites are bacterial derived and which are host derived. However, the observed decrease in many metabolites from multiple pathways implies that *STmΔaroA* utilize tumor-derived metabolites. WT *S*Tm have been reported to utilize succinate and lactate within the intestinal environment (27, 60), and we found a reduction of succinate in vitro only when live *STmΔaroA*, and not heat-killed, were present, suggesting active use of tumor metabolites by *STmΔaroA*. An interesting question raised by our observation of broad reductions in a range of metabolites within *STmΔaroA*-treated tumors is how this might affect immune responses. Several studies have demonstrated immune cell recruitment following BCT in xenograft/orthotopic models (9, 61, 62). Studies have shown dependence on innate immune subsets, such that depletion of monocytes or deficiency in key innate sensors such as MyD88 ablates therapeutic efficacy (63–65). In contrast, while some studies show the recruitment of adaptive cells, particularly CD8^+^ T cells (9), many studies have demonstrated treatment efficacy in Rag^–/–^ or nude mice, or upon CD4^+^ or CD8^+^ T cell depletion (5, 6, 8, 64, 66), indicating that adaptive immune responses are redundant. Whether the metabolic environment has a role in the apparent T cell redundancy is of interest. On one hand, we observe a reduction in key oncometabolites, such as 2-HG and lactate, which have known roles in immune suppression or promoting Tregs (67–70). On the other hand, the imposed metabolic competition for essential metabolic fuels, such as glucose or amino acids, may further impede T cell responses (71, 72). We would expect that *S*Tm acting as an adjuvant would induce immune cell recruitment, which would be important for maintaining tumor control. It will be of interest to determine whether the effect of metabolic competition by *S*Tm is a driver of T cell redundancy in these models and what impact that might have on long-term efficacy of BCT.

We show here that *STmΔaroA* can directly affect tumor environment and tumor cells and that this accounts — at least in part — for the efficacy of BCT. The effect of *S*Tm treatment of tumor organoids supports the hypothesis that BCT can directly affect the tumor, independently of effects via the immune system. Previous studies have shown that treatment of xenograft tumors with an AR-1–deficient *S*Tm strain could force tumor cells from G0/G1 to S/G2/M phase, sensitizing the tumor to chemotherapy with combined methioninase therapy (73, 74), thus also demonstrating a way by which *S*Tm treatment directly affects tumor cells. We provide evidence here of a reduction in tumor stemness characteristics as seen by reduced *Lgr5* and *Smoc2* in both CAC and *Apc^min/+^* models, as well as in tumor organoids derived from both models. We also found a slight increase in active caspase 3 following treatment in vitro*,* and using an Lgr5 reporter mouse, we found an overrepresentation of mCherry-*STmΔaroA* in Lgr5^+^ cells both in vivo and in vitro, with a majority of infected cells also appearing in the dead gate. Recent work by Fattinger et al., highlighted that *S*Tm infection was capable of inducing mixed cell death pathways in an epithelium-intrinsic manner (42). It is likely that, in our system, *S*Tm-mediated inflammasome activation also leads to heterogenous activation of cell death pathways, a process termed PANoptosis (75). Although outside of the scope of this study, it will be of interest to dissect/define the relative requirements for apoptotic, pyroptotic, and necroptotic cell death in the success of this therapy. It appears that *S*Tm treatment has a short-term effect on the ability of organoids to regrow. The reduction in stem cell transcripts and increase in cell death, particularly in Lgr5^+^ cells, would explain this delayed capacity to regrow. It is not surprising that the *STmΔaroA*-treated organoids recover; as they are passaged, the metabolic pressure that is imposed by *S*Tm is removed, so any surviving stem cells could repopulate the niche. However, given that we initially reseed organoids at the same density, it is likely that there are short-term transcriptional effects on the uninfected cells, possibly via the metabolic changes, which are eventually lost.

Our finding that just 1 or 2 doses of *STmΔaroA* can induce robust reduction in tumor burden (Figure 8), along with the disappearance of *S*Tm colonies over time (Supplemental Figure 1 and Figure 8) implies that the early and striking effect on the metabolic landscape, as well as preferential infection of stem cells by *STmΔaroA*, likely drive an initial antitumor effect of this therapy. Induction of an immune response is then likely to be important for eliciting longer-term and wider antitumor effects. It is important to note that colonization of tumors was characterized by large extracellular colonies, with some individual bacteria dispersed and infecting intracellularly. Only around 2%–7% of cells within a tumor were infected when analyzed by flow cytometry. Thus, it is implausible that *S*Tm will reach every tumor cell, or protumorigenic stromal cells of interest, to induce cell death and removal. Therefore, the effect of *S*Tm on the metabolic environment and then eliciting an immune response is critical for the success of BCT. This will be important when considering what tumor characteristics are further targeted by BCT.

Several groups are taking the approach of engineering bacteria to deliver drugs or other compounds that can further promote tumor death or immune clearance (63, 76–80). Given that bacteria home specifically to tumors, they are the ideal device to use to ensure tumor-specific drug targeting (3). The data we present here show that BCT does induce tumor regression in autochthonous models of cancer, and we show strong effects on the tumor metabolome and transcriptome. However, it is apparent that *STmΔaroA* alone does not cure the mice of intestinal tumors, so further engineering of the bacteria and/or cotherapies are required. By understanding the mechanisms of action, we could further improve the engineering of bacteria for BCT — for example, by delivering an engineered bacterium that can better utilize metabolites or by delivery of a cytotoxic compound that can further permeate through the tumor (81). Furthermore, rational selection of tumor types to be targeted, type of bacteria and attenuations, and delivery route are all likely to be important considerations for the success of BCT. This study demonstrates that CRC is an excellent candidate for targeting with BCT via oral delivery of attenuated *S*Tm.

## Methods

Detailed methodology can be found in the Supplemental Methods.

### Data availability.

RNA-Seq data are uploaded and available online (Gene Expression Omnibus, GSE136029).

### Statistics.

All data are presented as mean ± SD, unless otherwise indicated. One-way ANOVA, 2-tailed Students *t* test, or nonparametric statistical tests were used, as indicated for each figure, and were conducted using GraphPad Prism 8. *P* < 0.05 was considered statistically significant.

### Study approval.

All animal experiments were approved by the Institutional Animal Care and Use Committees of RIKEN Yokohama Branch (2018-1[3]) and Yokohama City University (T-A-17-001) or the University of Birmingham local animal welfare and ethical review body and authorized under the authority of Home Office license P06118734 (held by KMM).

## Author contributions

KMM conceived the project, designed and performed experiments throughout, analyzed data, and wrote the manuscript. GMM performed organoid experiments and helped write the manuscript. AC performed flow cytometry and helped write the manuscript. MT provided technical assistance throughout and maintained animal breeding. YN performed the GC-MS metabolomics experiments and analyzed data. IE performed organoid experiments. T Kato performed the microbiota 16s RNA-Seq and analysis. H Oda analyzed RNA-Seq data. T Kanaya generated mCherry and GFP-expressing *STmΔaroA*, helped establish organoid expertise, and sought local (RIKEN) experimental ethics authority. H Ohno advised on the project and provided funding.

## Supplementary Material

Supplemental data

## Version 1 10/28/2021

In-Press Preview

## Version 2 12/08/2021

Electronic publication
