# Supplementary material for: Bacterial cancer therapy in autochthonous colorectal cancer affects tumor growth and metabolic landscape
Source: JCI Insight. 2021 Oct 28;6(23):e139900. doi: 10.1172/jci.insight.139900 (PMC8675204; doi:10.1172/jci.insight.139900)
Supplement: Supplemental data [file jciinsight-6-139900-s017.pdf]

Supplementary Material

Materials and Methods

Animals

Animal studies at RIKEN, Japan: C57B/6J mice were purchased from JAX or CLEA (Japan). *Apc*$^{min/+}$ mice were purchased from JAX (https://www.jax.org/strain/002020). Lgr5-GFP reporter mice were perchased from JAX (https://www.jax.org/strain/008875). Heterozygous mice harbour an Lgr5-EGFP-IRES-creERT2 "knock-in" allele that both abolishes Lgr5 gene function and expresses EGFP and CreERT2 fusion protein. When these mice are bred with mice containing a loxP -flanked sequence of interest, tamoxifen-inducible, Cre-mediated recombination will result in deletion of the floxed sequences in the <i>Lgr5</i>-expressing cells of the offspring. Animal breeding was conducted under specific pathogen-free (SPF) conditions at the RIKEN Center for Integrative Medical Sciences Animal Facility and infection experiments were conducted at the conventional facility at Yokohama City University. 12 hr day/night cycles (7am-7pm in both facilities) and chow and water were fed *ad libitum*. For *Apc*$^{min/+}$ mice, litters were randomly assigned into treatment groups and both male and female mice were used. Animal studies at University of Birmingham, UK: C57B/6J were purchased from Charles River and *Apc*$^{min/+}$ mice (JAX) were maintained within the Biomedical Services Unit (BMSU), UoB, under SPF conditions. All experiments were performed within the procedural floors of the BMSU. 12 hr day/night cycles and chow and water were fed *ad libitum*. Male and female mice were used as indicated in figure legends. For *Apc*$^{min/+}$ mice, litters were randomly assigned into treatment groups and both male and female mice were used.

Colitis-associated cancer model

C57B6/J mice were purchased at 6 weeks of age and allowed settle into the mouse facility for 1-2 weeks. Starting at 7 to 8 weeks of age, mice were given one i.p. injection of AOM (10 mg/kg, Sigma) in the afternoon. The following day, mice were given DSS (1%, MW 35 000-50 000, MP Biochemicals) in the drinking water for 5 days, followed by 16 days normal water. 2 more doses of DSS (1%) were given for 5 day periods. After the 3rd DSS dose, mice recovered for 1 to 2 weeks before starting the *STm*$^{\Delta aroA}$ treatment.

Apc$^{min/+}$ model

Wild-type C57B/6J female mice were bred with heterozygote *Apc*$^{min/+}$ males. Litters were weaned at 4 weeks of age and genotype confirmed. *Apc*$^{min/+}$ mice (male and female) were transferred to the experimental facility at 6 weeks of age, and *STm*$^{\Delta aroA}$ treatment started at 8 weeks. At the end of the protocol, genotypes were re-confirmed.

STm$^{\Delta aroA}$ treatment

UF020 *Salmonella* typhimurium strain lacking aromatase A (1) was grown overnight in LB broth with ampicillin (200 ug/mL). In the morning a 1:20 subculture was grown in LB broth (no ampicillin) to an OD_{600} of 0.7 to 0.8 OD_{600} = 5x10^5 CFU/mL). Cultures were centrifuged (4000 rpm, 8 mins, room temperature) and washed twice in PBS before resuspending in PBS at 5x10^9 CFU per 100 uL. This was transferred to the animal facility within 30 minutes and mice were delivered 100 uL by oral gavage at the indicated time points.

Tissue isolation and analysis

At the end of the $STm^{\Delta aroA}$ treatment, mice were killed and colons (CAC) or SI ($Apc^{min/+}$) were dissected out, cut longitudinally and washed thoroughly in cold PBS. Tumors were counted under a stereo microscope using an eyepiece graticule with 10x10 mm grid. Tumors and normal tissue were then micro-dissected and either snap frozen in liquid N_2 for later analysis or used for assessing bacterial colonization (CFU).

Colony forming unit analysis

Tissue was homogenized in PBS containing 0.1% triton-X using a hand-held homogenizer. After serial dilution, 50 uL was plated onto LB agar plates containing ampicillin (200 ug/mL) and incubated at 37°C overnight. Colonies were counted and CFU calculated per gram of tissue.

Scanning electron microscopy

Colons were dissected out, cut longitudinally and thoroughly washed in PBS. Sections around 1 cm were cut and fixed in 2.5% glutaraldehyde in 0.1% phosphate buffer for 24 hours at 4°C. Tissue was then dehydrated in increasing concentration of ethanol (50% -> 70% -> 80% -> 90% -> 95%-> 100% -> 100%) for 15 minutes each, then tissues substituted with t-butyl alcohol followed by freeze drying. Before mounting samples, tumors were cut on the sagittal plane to reveal the tumor core then mounted onto aluminum stubs and were metal-coated using a magnetron sputter (MSP-1S; Vacuum Device), and examined by scanning electron microscopy (VE-7800; Keyence).

DNA extraction and 16S rRNA sequencing

Mouse colonic content was collected and stored at -80°C until ready for analysis. Fecal DNA extraction was performed according to the literature (2–4). DNA from fecal samples was extracted by the method using lysozyme (Wako), achromopeptidase (Wako) and proteinase K (Merck Japan). Bacterial DNA from feces was amplified by PCR as previously described (4). PCR primers (V4 region of 16S rRNA sequence: 515F and 806R) with adaptor sequencing for Illumina miseq platform were used: the forward primer (5'-AATGATACGGCGACCACCGAGATCTACAC NNNNNNNN TATGGTAATTGTGTGCCAGCMGCCGCGGTAA-3') and reverse primer (5'-CAAGCAGAAGACGGCATACGAGAT NNNNNNNN AGTCAGTCAGCCGGACTACHVGGGTWTCTAAT-3') (5). The "NNNNNNNN" sequence unique to each samples was attached into primer for multiplexing.

PCR amplification using Ex Taq HS (Takara) was performed for 25 cycles with the condition as previously described (4). Amplified PCR products were purified using AMPure XP (Beckman Coulter). DNA concentration of each sample was measured using Quant-iT PicoGreen ds DNA Assay Kit (Life Technologies). Mixed samples were prepared by pooling approximately equal amounts of PCR amplicons from each sample. The DNA library with 20% denatured PhiX spike-in was sequenced by Miseq sequencer using 500 cycles kit (Illumina).

Taxonomic assignments and estimation of relative abundance of sequencing data were performed using the analysis pipeline of the QIIME software package (Qiime version 1.9.1) (6). Chimera checking was performed using UCHIME (7). OTUs were clustered at 97% similarity. We performed taxonomy assignment against Greengenes database version 13.8 using RDP classifier (8,9). Alpha diversity and beta diversity were assessed

by calculating Chao1 index, Shannon diversity index, Simpson's diversity index and weighted Unifrac distance for each sample at equal sequencing depths (10,000 reads/ sample). We have visualized the beta diversity using principal coordinates analysis (PCoA) of weighted UniFrac distance between samples (10).

RNA isolation

RNA was isolated using Qiagen RNeasy Mini kits, as per manufacturer instructions. Briefly, tissue stored in -80°C was placed in buffer RLT + β-mercaptoethanol and homogenized by bead beating. Lysates were then processed as recommended, with the optional DNase digest. RNA was quantified using a DeNovix DS-11.

cDNA library prep and RNA sequencing

RNA integrity was assessed by Agilent Bioanalyzer before proceeding RIN values of 8 or greater were used. 2 ug of RNA was used to prepare a cDNA library using TruSeq RNA Library Prep Kit v2 (Illumina). Sequencing was performed on an Illumina HiSeq 1500 System in a 1 x 50 bp single read mode. Sequenced reads were mapped against the mouse reference genome (mm10) using TopHat (11), and gene expression was quantified by Cufflinks (12). Gene ontology enrichment analysis was performed using DAVID (13,14). The original RNAseq data is uploaded and available online (Gene Expression Omnibus: GSE136029).

cDNA prep and qPCR

cDNA was prepared using standard oligoDT and M-MLV reverse transcriptase. Quantitative real-time PCR was performed with the LightCycler® 480 Real-Time PCR System (Roche) and SYBR Premix Ex Taq (Takara). Gene-specific primers (Eurofins Genomics, Japan) are listed in **Table S1**.

Immunofluorescence

Freshly dissected colon tissue was swiss-rolled and placed in 4% PFA overnight. Samples were paraffin embedded and 5 uM sections cut. Paraffin sections were rehydrated and washed with PBS. Samples were boiled in citrate buffer for 15 minutes for antigen retrieval. Then incubated with 1% BSA/PBS supplemented with 5% normal serum donkey serum to quench the nonspecific binding of antibodies. Goat anti-E-cadherin (R&D Systems, 1:200) and Ki67 antibody (1:200; CST) was incubated overnight, washed, secondary stain with anti-goat IgG or anti-rabbit IgG (LifeTechnologies) and counterstained with DAPI. Samples were imaged using an Axiovert Slidescanner (Zeiss) and images processed using Zen software (Zeiss).

Flow cytometry

Ex vivo: Tumors were excised from Lgr5-GFP CAC-induced mice that had been treated for 24hours with mCherry-STm$^{\Delta aroA}$. Tumors were minced into small pieces and incubated with 5 mL digestion buffer (DMEM with 2.5% FBS, ampicillin, 200U/mL collagenase D and DNAseI (2ml/mL) for 30 mins at 37°C with shaking. Supernatant was then collected into a 50 mL Falcon through a 70 μm filter and cells centrifuged at 1200 rpm for 5 mins. Cells were washed with PBS/2%FBS and filtered then transferred to a 96-well round bottom plate in PBS/FBS for staining. Cells were stained with viability dye eBioscience efluor506 (cat # 65-0866). EpCAM (eBioscience, APC, cat# 17-5791-82, clone G8.8, 1:400). WT splenocytes were taken for compensation against the Lgr5-GFP reporter.

In vitro: Tumor organoids derived from Lgr5-GFP CAC-induced mice were grown and infected with mCherry-expresssing STm$^{\Delta aroA}$ as described below. Following 24 hrs infection organoids were recovered from Matrigel

using Cell Recovery Solution (BD), spun at 1200 rpm for 5 mins then resuspended in 500 µL TrypLE with ROCK inhibitor (Y27632, 10mM used at 1:500) and mixed by pipetting and incubated in a 37°C water bath for 3 mins with regular agitation. Another 500 µL of basic medium (see Table 2) with ROCK inhibitor (1:500) was added and organoids thoroughly pipetted to dissociate using a 10 µL tip on the end of a 1 mL tip. These were then spun before resuspending in FACS buffer (PBS/2%FBS) and stained in a 96-well plate (as above but excluding EpCAM).

GC-MS analysis of metabolites

Extraction and measurement of metabolites were previously described (15) with some modifications. Tumor and normal intestinal tissues and tumor organoids (approximately 10 mg) were added to 125 µl methanol, 150 µl Milli-Q water containing internal standard (100 µmol/l 2-isopropylmalic acid) and 60 µl CHCl3 and disrupted with zirconia beads using Micro Smash MS-100 (Tomy Seiko). All samples were shaken at 1,200 rpm for 30 min at 37°C. After centrifugation at 16,000 × g for 5 min at room temperature, 250 µl of the supernatant were transferred to a new tube and 200 µl of Milli-Q water added. After being mixed, the solution was centrifuged at 16,000 × g for 5 min at room temperature, and 250 µl of the supernatant were transferred to a new tube. Samples were evaporated for 20 min at 40°C, and then lyophilized using a freeze dryer. Dried extracts were firstly methoxymated with 40 µl of 20 mg/ml methoxyamine hydrochloride (Sigma-Aldrich) dissolved in pyridine. After adding the derivatization agent, samples were shaken at 1,200 rpm for 90 min at 30°C. Samples were then silylated with 20 µl of N-methyl-N-trimethylsilyl-trifluoroacetamide (GL Science) for 30 min at 37°C with shaking at 1,200 rpm. After derivatization, samples were centrifuged at 16,000 × g for 5 min at room temperature, and the supernatant transferred to glass vial for gas chromatography-tandem mass spectrometry measurement using a GCMS-TQ8030 triple quadrupole mass spectrometer (Shimadzu) with a capillary column (BPX5, SGE Analytical Science). The GC program was previously described (15). Data processing was performed using LabSolutions Insight (Shimadzu).

The quantified metabolome data was statistically analyzed using Orthogonal Partial Least Square-Discriminant Analysis (OPLS-DA) with SIMCAP+ software (Version 12.0.1.0, Umetrics, Umeå, Sweden) using UV scaling method. Potential metabolites were selected based on the Variable Importance in Projection (VIP) score greater than 1.0 and uploaded to MetaboAnalyst 3.0 (http://www.metaboanalyst.ca) for pathway analysis. The functional pathway analysis of potential biomarkers was based on the database source of the Kyoto Encyclopedia of Genes and Genomes (http://www.genome.jp/kegg/).

Tumor organoid establishment and culture

Tumor organoids were established as previously described (16), with some alterations. Buffers and culture medium components are listed in **Table S2**. Tumors were dissected from the colons of mice that had CAC-induced tumors and from the SI and colon of $Apc^{min/+}$ mice. Tumors were washed in cold PBS then incubated in 10 mL chelation buffer with 2mM EDTA for 60 mins on ice. Tubes were shaken vigorously by hand (removes most normal epithelium) and EDTA buffer was removed and tumor tissue then washed 3x with chelation buffer. Tissue was then incubated in digestion buffer for 30 minutes at 37°C with shaking. Supernatant was filtered through a 70 µM filter, remaining tissue wash once with basic medium and also filtered. Cells were then pelleted (300 g for 3 minutes) washed once then resuspended in 100-200 uL Matrigel (Corning 356231) (50 uL per well) and cultured in 24-well plate with 500 µL complete medium. Once established for 1 to 2 weeks, cultures were switched to EGF-only medium.

For maintenance, organoids were split every week. In brief, all tubes and tips were pre-coated with FBS, organoids were manually disrupted from the Matrigel using P500 pipette and transferred to a 50 mL Falcon tube. Tubes were centrifuged at 300 g for 5 mins at 4 deg. Excess medium was removed carefully to not removed Matrigel containing the cell pellet. Organoids were resuspended in 4 mL Basic medium, transferred to a 15 mL tube and pipetted thoroughly using a fire-polished glass pipette to break up the organoids. These were centrifuged and resuspended in Matrigel and plated out. Split ratios of around 1:4 to 1:6 depending on density.

<u>$STm^{\Delta aroA}$ infection of tumor organoids</u>

Tumor organoids were infected at day 5 post-split to ensure good organoid size and integrity. $STm^{\Delta aroA}$ was grown overnight and sub-cultured as described above. 5 µL containing 1×10^8 CFU was dropped into the culture medium (or PBS control) and left for 2 hours to allow for bacterial invasion of the Matrigel and organoids. After 2 hours medium was removed and Matrigel washed 2x with PBS and medium replaced and gentamycin added. These were cultured overnight and organoids collected 24 hours after initial infection for analysis. For qPCR analysis, buffer RLT was added directly to the culture plate (after removing medium and washing with PBS) which completely dissolved the Matrigel, these were then processed for RNA isolation as described above. 1 well was used for each technical replicate. For metabolome and succinate assays, culture medium was removed and the Matrigel washed with PBS. BD Cell Recovery Solution was added and plate kept on ice for 1 hr to dissolve the Matrigel. Organoids were collected into Eppendorf tubes spun and washed twice with cold PBS. Organoids were then snap frozen and stored at -20 until analysis.

Heat killed $STm^{\Delta aroA}$ was prepared by incubating at 95°C for 5 minutes. Effective killing was tested by plating out on LB agar. SN was prepared by growing $STm^{\Delta aroA}$ in tumor culture medium until an OD_{600} of approximately 0.7. SN was filtered with a 10 kDa cut-off columns. 1×10^5 heat-killed $STm^{\Delta aroA}$ were used as this is the average CFU count obtained from the live infections after 24 hours, and the amount of SN added was also calculated based on this CFU.

<u>Stem-forming Assay</u>

Organoids were grown and infected as described above. After 24 hours, Matrigel was digested with CellRecovery Solution (BD) for 1 hour at 4°C. Organoids were transferred to a 15 mL tube, spun at 300g for 5 mins, supernatant removed and 1 mL of TrypLE added. Tubes were placed in a water bath at 37°C and shaken every minutes for approximately 4 minutes. Cells were then pipetted with a fine 10uL tip to aid dissociation, washed and resuspended for counting. Equal numbers of organoids (20-50 000, depending on experiment) were then resuspended in Matrigel and plated out. Following passages were done as per maintenance (with thorough pipetting of organoids without TrypLE) and were reseeded at equal split ratio (eg 1:12).

<u>Succinate Assay</u>

Succinate assay (Sigma) was performed on cell lysates as per the manufacturers protocol and 96-well plates were measured on a spectrophotometer (Spectrostar Nano – BMG Labtech) at the respective wavelengths.

<u>MTT Assay</u>

Following dissociation of organoids for the stem-forming assay (above), 4, 000 cells were re-seeded per well into a flat-bottom 96-well plate in 10 µL Matrigel with150 µL tumor medium, with each sample seeded in

triplicate (results averaged). At the indicated timepoints, medium was aspirated and replaced with 50 µL tumor medium and 50 µL MTT reagent (AbCAM) and then incubate at 37 °C for 1 – 3 hrs. The reaction was stopped when visible purple formazan crystals were formed by addition of MTT solvent (4 mM HCl, 0.1% NP40 in isopropanol). The plate was measured on a plate-reader at OD560 (BioRAD).

Caspase3 activity Assay

A colorimetric caspase-3 activity assay kit (Sigma) was used. Organoids were infected with $STm^{\Delta aroA}$ or control as outlined above. After 24 hrs, organoids were recovered from Matrigel using CellRecovery Solution (BD) and the cell pellet spun in Eppendorf tubes and resuspended in 100 µL of the kit lysis solution. Cell lysates were transferred to a flat bottom 96 well plate and assay performed as per manufacturer instructions.

LDH Assay

Cell culture supernatants from organoids treated for 24 hours (as indicated above) were collected into Eppendorf's and spun at top speed for 5 mins at 4°C to remove any debris. 50 µL of SN from each sample was added to a 96 well plate in triplicate and 50 µL of LDH assay solution (made according to manufacturer instructions, ThermoFisher) was added. The plate was incubated at RT, in the dark, for 30 mins or until the color had developed. 50 µL of stop solution was added and plate was measured on a plate reader (BioRAD) as instructed. For the positive control, a well of organoids were treated with the provided cell lysis solution for 30 minutes at 37 °C and the SN collected. Medium alone served as the background control. % cell death was calculated as follows (after removing background absorbance): sample OD/+ve control OD *100. An average of sample triplicates were plotted.

Statistical analysis

All data is presented as mean +/- SD. One-way ANOVA, Students T-test or non-parametric statistical tests were used, as indicated for each figure, and were conducted using GraphPad Prism 8. $P<0.05$ was considered statistically significant.

Table S1: Primers used for qPCR analysis

Gene name	Sequence fwd	Sequence rev	Species
Pdk4	CCGCTGTCCATGAAGCA	GCAGAAAAGCAAAGGACGTT	mouse
Twist	CGGGTCATGGCTAACGTG	CAGCTTGCCATCTTGGAGTC	mouse
Snail	CCACACTGGTGAGAAGCCATTC	TCTTCACATCCGAGTGGGTTT	mouse
Smoc2	CCCTCAGAAGCCACTCTGTG	ACTTGCTGGAACTCCTTCCG	mouse
Vim (Vimentin)	CGGAAAGTGGAATCCTTGCA	CACATCGATCTGGACATGCTGT	mouse
Ccnd1 (CyclinD1)	ACCTGCATGTTCGTGGCCTCTAAG	CTCATCCGCCTCTGGCATTTTG	mouse
Lgr5	CGGAGGAAGCGCTACAGAAT	CTGGGTGGCACGTAGCTGAT	mouse
Gapdh	TGTGTCCGTCGTGGATCTGA	TTGCTGTTGAAGTCGCAGGAG	mouse
18s	GATCCATTGGAGGGCAAGTCT	CCAAGATCCAACTACGAGCTTTT	mouse

Table S2: Intestinal tumor organoid culture reagents

**Chelation buffer**	in distilled water:<br>- 5.6 mmol/L Na2HPO4<br>- 8.0 mmol/L KH2PO4<br>- 96.2 mmol/L NaCl<br>- 1.6 mmol/L KCl<br>- 43.4 mmol/L sucrose<br>- 54.9 mmol/L D-sorbitol<br>- 0.5 mmol/L DL-dithiothreitol (added fresh)
**Digestion buffer**	DMEM:<br>- 2.5% FBS<br>- pen/strep<br>- 400U/mL collagenase D<br>- 25 U/mL Dispase
**Basic medium**	- Advanced DMEM F12 – 500mL (Life Technologies)<br>- Pen/Strep 1:100<br>- Glutamax – 1:100<br>- HEPES 1:100
**2xN2/B27/NA medium**	- N2 (200 uL of 100x)<br>- B27 (400 uL of 50x)<br>- NA (50 uL of 500 mM)<br>- Added to 10 mL of Basic medium – use within 1-2 weeks
**Complete medium**	- Basic medium + 2xN2/B27/NA (1:1)<br>- mNoggin. Peprotech Cat#250-38. 1000x stock: 100 ug/mL in 0.1% BSA/PBS. 20 uL aliquots. Store at -20.<br>- mEGF. Peprotech Cat#315-09. 10,000x stock: 500 ug/mL 0.1% BSA/PBS. 5uL aliquots. *before use, add 45uL basic medium to make 1000x solution.<br>- Rspol conditioned medium (CM) 1:20<br>- CHIR99021 (GSK3 inhibitor). 5mM in DMSO.<br>- Y-27632 (ROCK inhibitor). 10mM in PBS. Use only for recovering frozen stock, or first establishing.<br>Make as you need it, use immediately
**Tumor medium**	- Basic medium + 2xN2/B27/NA (1:1)<br>- mEGF (1:1000)<br>- ROCK inhibitor (Y27632) 1:500 for recovery (not in growth)<br>Make as you need it, use immediately

Supplementary References:

1. Gulig PA, Doyle TJ. The Salmonella typhimurium virulence plasmid increases the growth rate of salmonellae in mice. Infect Immun. 1993 Feb;61(2):504–11.
2. Kim SW et al. Robustness of gut microbiota of healthy adults in response to probiotic intervention revealed by high-throughput pyrosequencing. DNA Res. 2013 Jun 1;20(3):241–53.
3. Morita H et al. An Improved DNA Isolation Method for Metagenomic Analysis of the Microbial Flora of the Human Intestine. Microbes Environ. 2007;22(3):214–22.
4. Kato T et al. Oral Administration of Porphyromonas gingivalis Alters the Gut Microbiome and Serum Metabolome. mSphere. 2018 Oct 17;3(5).
5. Kozich JJ et al. Development of a dual-index sequencing strategy and curation pipeline for analyzing amplicon sequence data on the miseq illumina sequencing platform. Appl Environ Microbiol. 2013 Sep 1;79(17):5112–20.
6. Caporaso JG et al. QIIME allows analysis of high-throughput community sequencing data. Nat Methods. 2010 May 11;7(5):335–6.
7. Edgar RC et al. UCHIME improves sensitivity and speed of chimera detection. Bioinformatics. 2011 Aug 15;27(16):2194–200.
8. McDonald D et al. An improved Greengenes taxonomy with explicit ranks for ecological and evolutionary analyses of bacteria and archaea. ISME J. 2012 Mar 1;6(3):610–8.

9. Wang Q et al. Naïve Bayesian classifier for rapid assignment of rRNA sequences into the new bacterial taxonomy. Appl Environ Microbiol. 2007 Aug 15;73(16):5261–7.
10. Lozupone C, Knight R. UniFrac: A new phylogenetic method for comparing microbial communities. Appl Environ Microbiol. 2005 Dec;71(12):8228–35.
11. Kim D et al. TopHat2: Accurate alignment of transcriptomes in the presence of insertions, deletions and gene fusions. Genome Biol. 2013 Apr 25;14(4):R36.
12. Trapnell C et al. Differential analysis of gene regulation at transcript resolution with RNA-seq. Nat Biotechnol. 2013 Jan 9;31(1):46–53.
13. Huang DW, Sherman BT, Lempicki RA. Bioinformatics enrichment tools: Paths toward the comprehensive functional analysis of large gene lists. Nucleic Acids Res. 2009 Jan 1;37(1):1–13.
14. Huang DW, Sherman BT, Lempicki RA. Systematic and integrative analysis of large gene lists using DAVID bioinformatics resources. Nat Protoc. 2009 Jan 18;4(1):44–57.
15. Sato Y et al. Maternal gut microbiota is associated with newborn anthropometrics in a sex-specific manner. J Dev Orig Health Dis. 2019 May 20;10(6):659–66.
16. Sato T et al. Long-term expansion of epithelial organoids from human colon, adenoma, adenocarcinoma, and Barrett's epithelium. Gastroenterology. 2011/09/06. 2011;141(5):1762–72.

Supplementary Figures

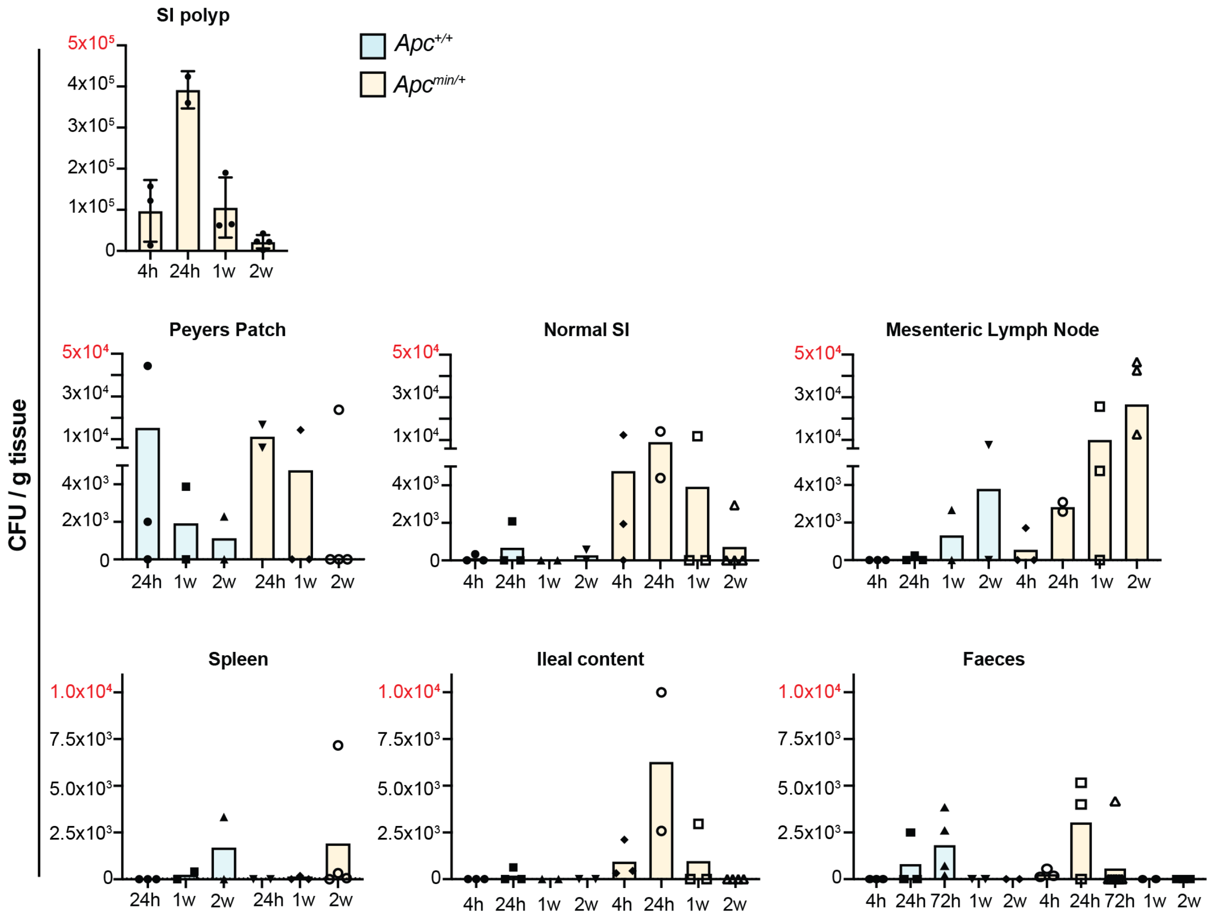

Figure S1: CFU in peripheral organs and SI 24 hours after *STm*$^{\Delta aroA}$ treatment
Apc$^{min/+}$ and *Apc*$^{+/+}$ mice were given 5x10^{9} CFU of *STm*$^{\Delta aroA}$ by oral gavage and bacterial colonization was assessed 4 hours (not all tissues), 24 hours, 1 week and 2 weeks later. An additional timepoint of 72 hours for feces analysis was also conducted (taken from mice for the 1 and 2 week timepoints) . Spleen, mesenteric lymph nodes, Peyer's patches, normal small intestinal (SI) tissue and SI tumors were dissected and Ileal contents and feces collected, homogenized in PBS – 0.1% triton-X and plated onto LB agar plates containing ampicillin. Each dot represents one mouse.

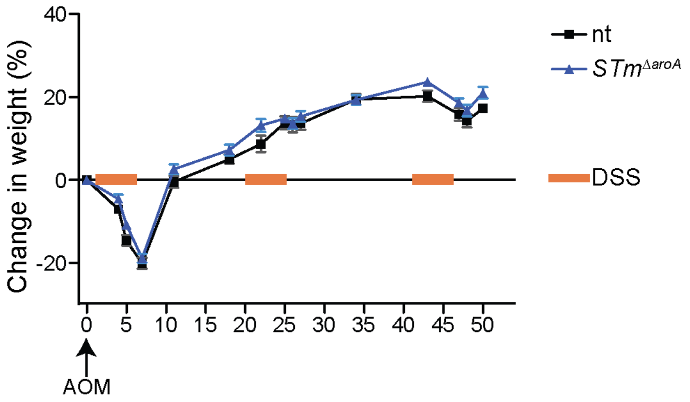

Figure S2. Mouse weight change during AOM/DSS CAC induction
Weight change in mice from Figure 1B. Mice were treated with AOM and DSS (see Figure 1A and methods) to induce colitis-associated cancer. Mouse weight was monitored over the induction period and mice allocated to treatment or non-treatment groups after the last dose of DSS, assuring similar DSS-induced weight change between groups.

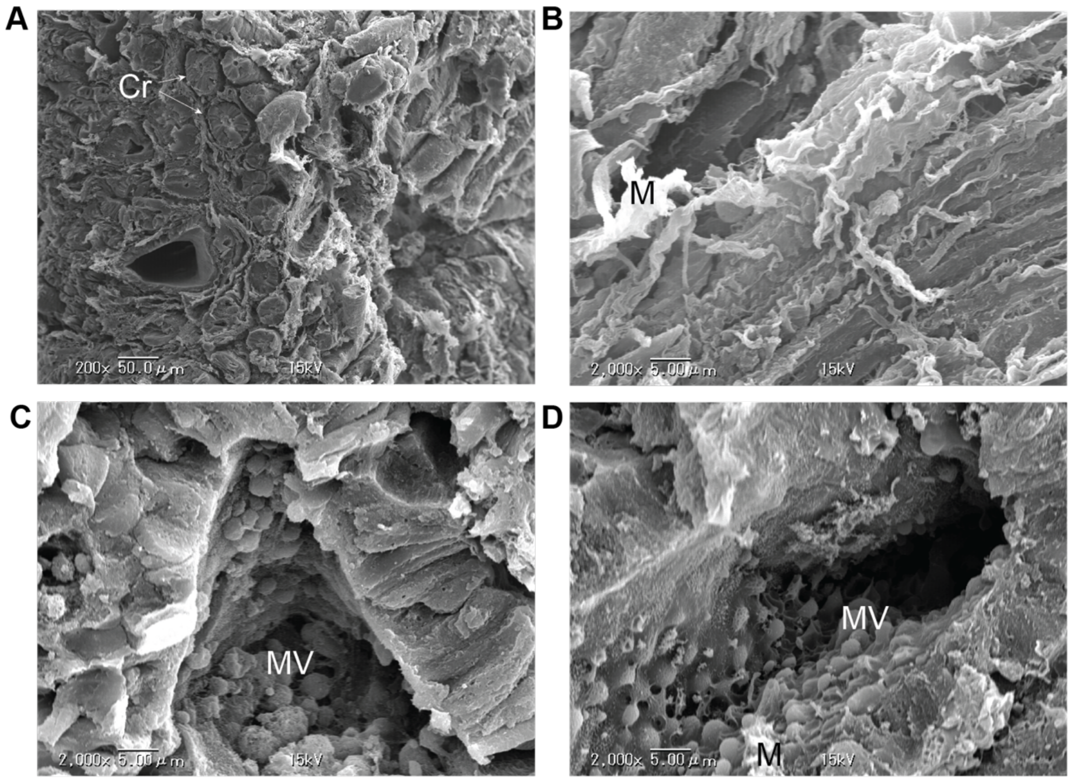

Figure S3: Scanning electron microscopy of tumors.
Mice bearing CAC colon tumors were given control vehicle by oral gavage and tissues were taken 24 hours later and prepared for SEM. (**A-C**) Vehicle control-treated tumors (**D**) STm-treated tumor. Cr = crypt, M = mucous, MV = microvilli.

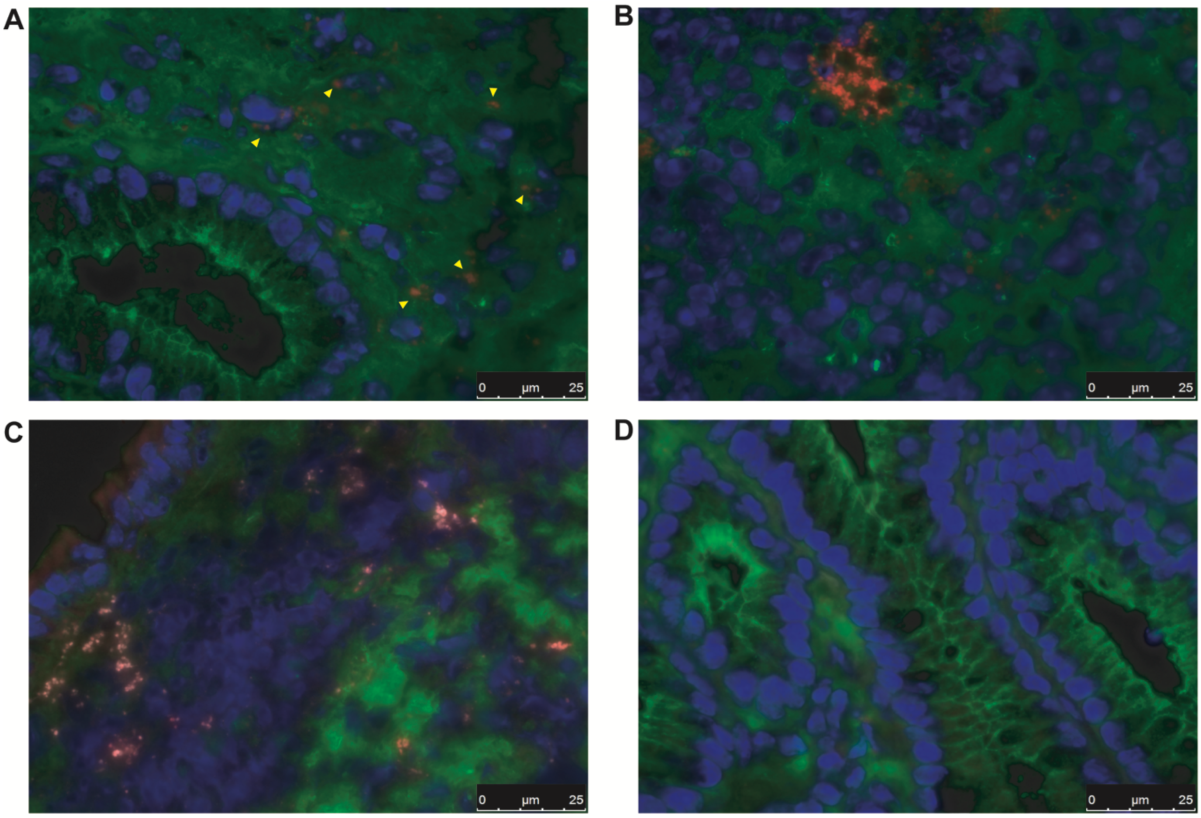

Figure S4: Immunofluorescent staining of STm within tumors.
Mice were treated with AOM and DSS followed by 6 weeks treatment with vehicle control or mCherry-expressing *STm*$^{\Delta aroA}$ (as per Figure 1A). Formalin fixed, paraffin embedded tissue sections were prepared for IF. Red = *STm*$^{\Delta aroA}$, Green = F-actin, Blue = DAPI. For reference of tissue orientation, **A** is in a similar localization to that indicated in Fig S5E (green boxes), **B** to Fig S5F, **C** to Fig S5G and **D** to Fig S5H. This indicates only approximate tissue localisation in relation to structures (such as lamina propria, immune infiltrate, epithelial barrier) not necessarily from same tissue section.

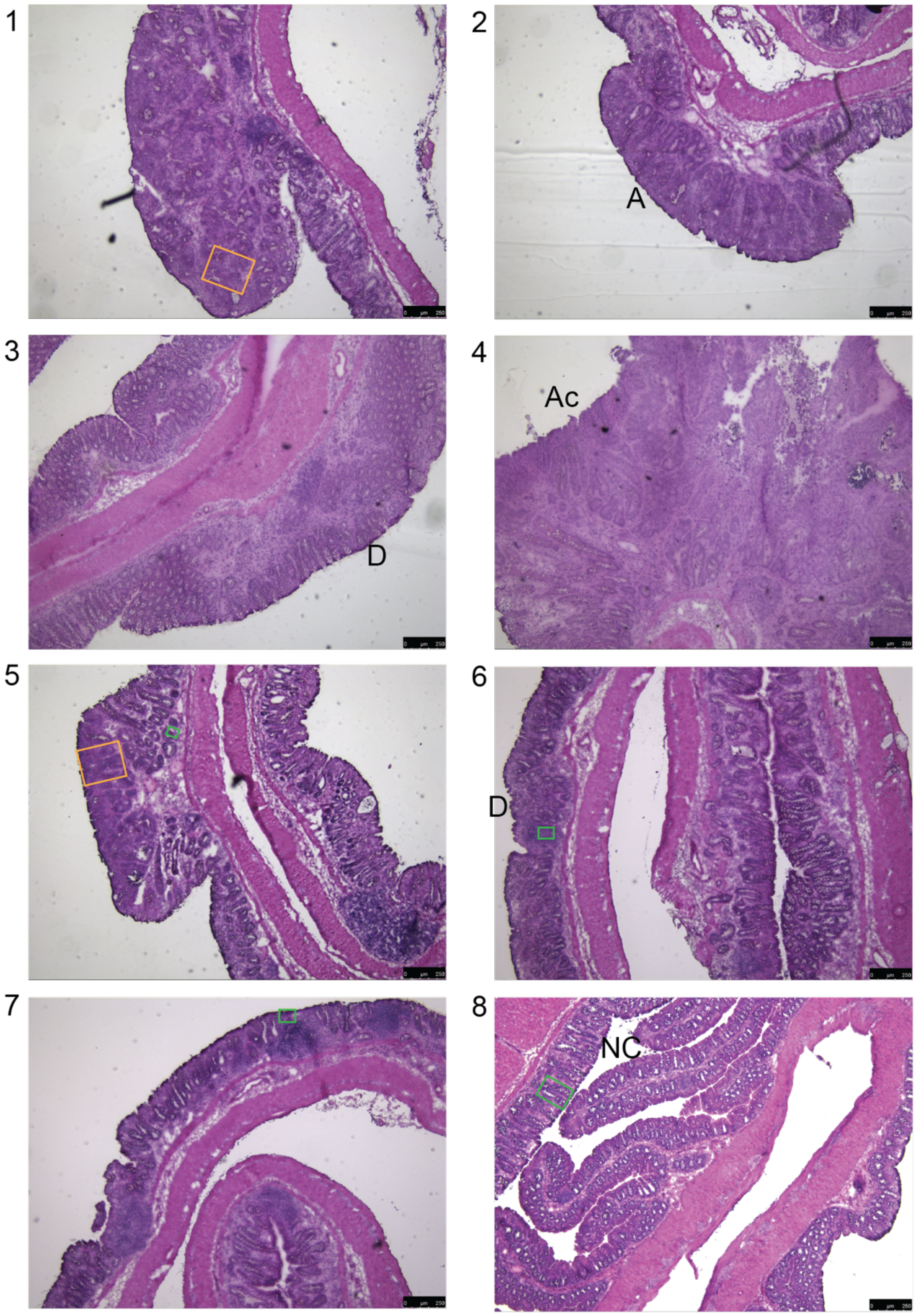

Figure S5: Histological appearance of CAC-induced mouse colon.
Mice were treated with AOM and DSS followed by 6 weeks treatment with vehicle control or mCherry-expressing STm$^{\Delta aroA}$ (as per Figure 1A). Formalin fixed, parrafin embedded tissue sections were prepared for haematoxylin and eosin (H&E) staining. 1 -4 are representative images from 4 non-treated mice and 5 - 8 are from STm$^{\Delta aroA}$-treated mice. MM = Muscularis mucosa, M = Muscularis, SM = sub-mucosa, Mu = Mucosa, Cr = Crypt, A = Adenoma, Ac = Adenocarcinoma, D = Dysplasia, NC = Normal crypts. Green boxes in 5-8 indicate the type of region imaged in Fig S4 and yellow boxes in 1 and 5 indicate the type of region imaged in Fig 4C.

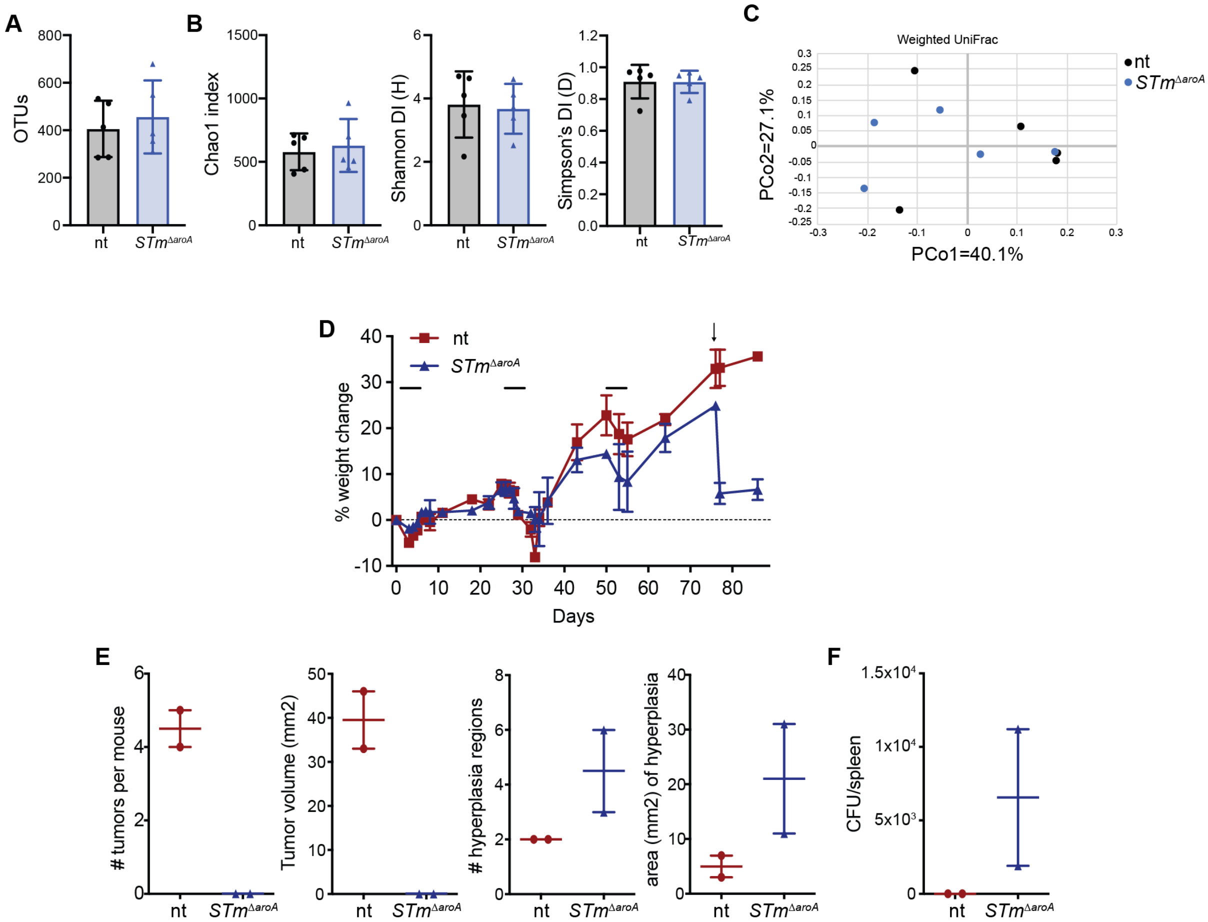

Figure S6: Microbiota are not involved in efficacy of BCT

A-C16s rRNA sequencing of colon contents of AOM/DSS-induced mice that had received 6 weeks *STm*$^{\Delta aroA}$, or control, treatment. Total OTU's were not different between treatment groups (**A**). Analysis of species alpha diversity and richness using multiple tests also showed no difference in the microbiota (**B**). Beta diversity measured by weighted UniFrac shows no difference in the quantitative abundance of microbial species (**C**). **D-G** Germ-free mice were treated with AOM and DSS to induce colorectal tumors, then treated by oral gavage with *STm*$^{\Delta aroA}$ (11 days treatment). (**D**) shows weight change over the period of tumor induction and following *STm*$^{\Delta aroA}$ administration. (**E**) tumor burden and tumor volume. (**F**) CFU in spleens.

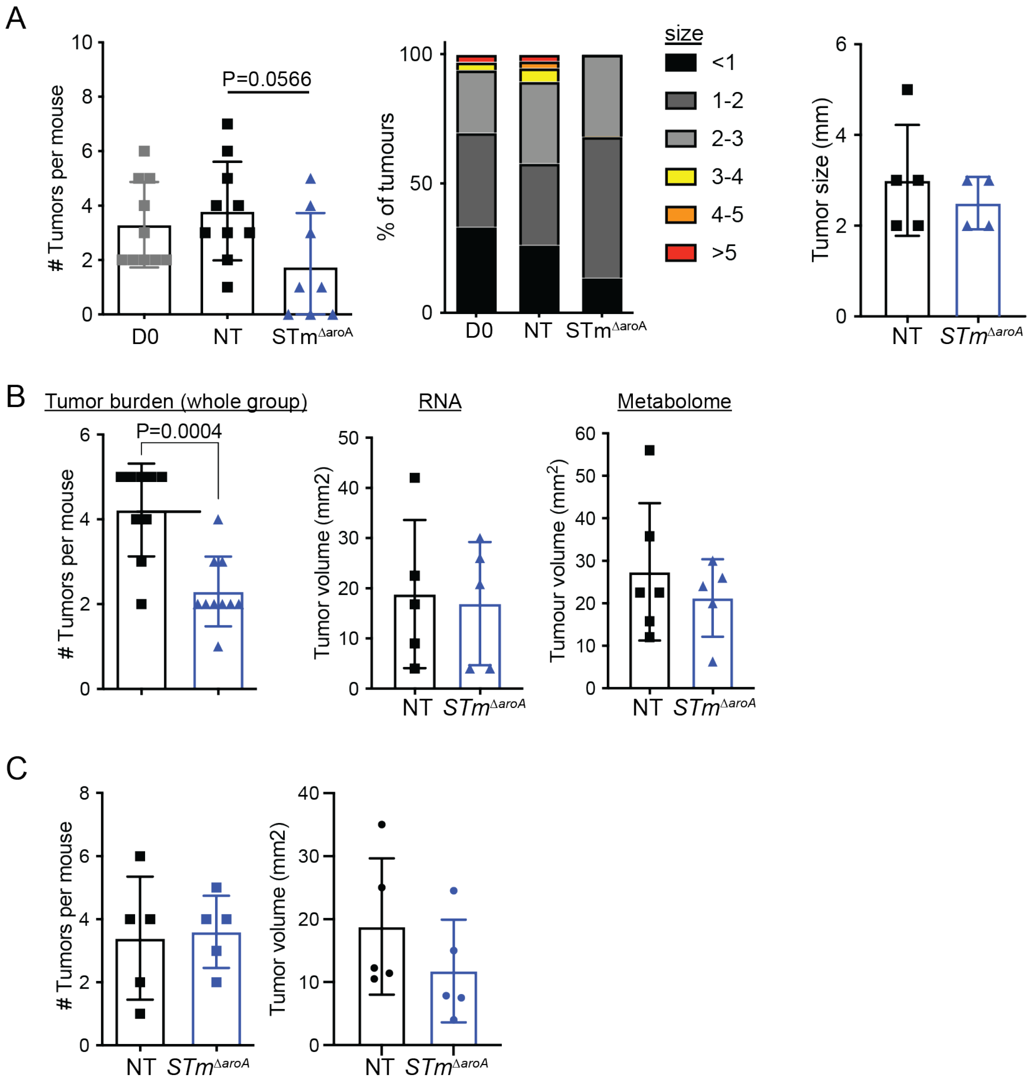

Figure S7: Size of tumors used in RNAseq, qPCR and metabolomics data aquisition. **A**) Shows the tumour burden (left) and tumour size (right) for each group in AOM/DSS induced mice treated for 4 weeks and used for RNAseq analysis. The right-hand-side graph show the individual size of the tumors RNA was isolated from. **B**) Shows the tumour burden (left) in AOM/DSS induced mice treated for 6 weeks with control or STmΔaroA. Middle graph shows the individual tumor volume of those tumors used for RNA extraction and downstream qPCR analysis, and the right-hand-side graph shows the individual tumor volume for those used for metabolite extraction and analysis. **C**) Shows the tumor burden in AOM/DSS-induced mice treated for 24 hours with control or *STm*$^{\Delta aroA}$ (left) and the individual volume of tumors used for 24 hr metabolome analysis.

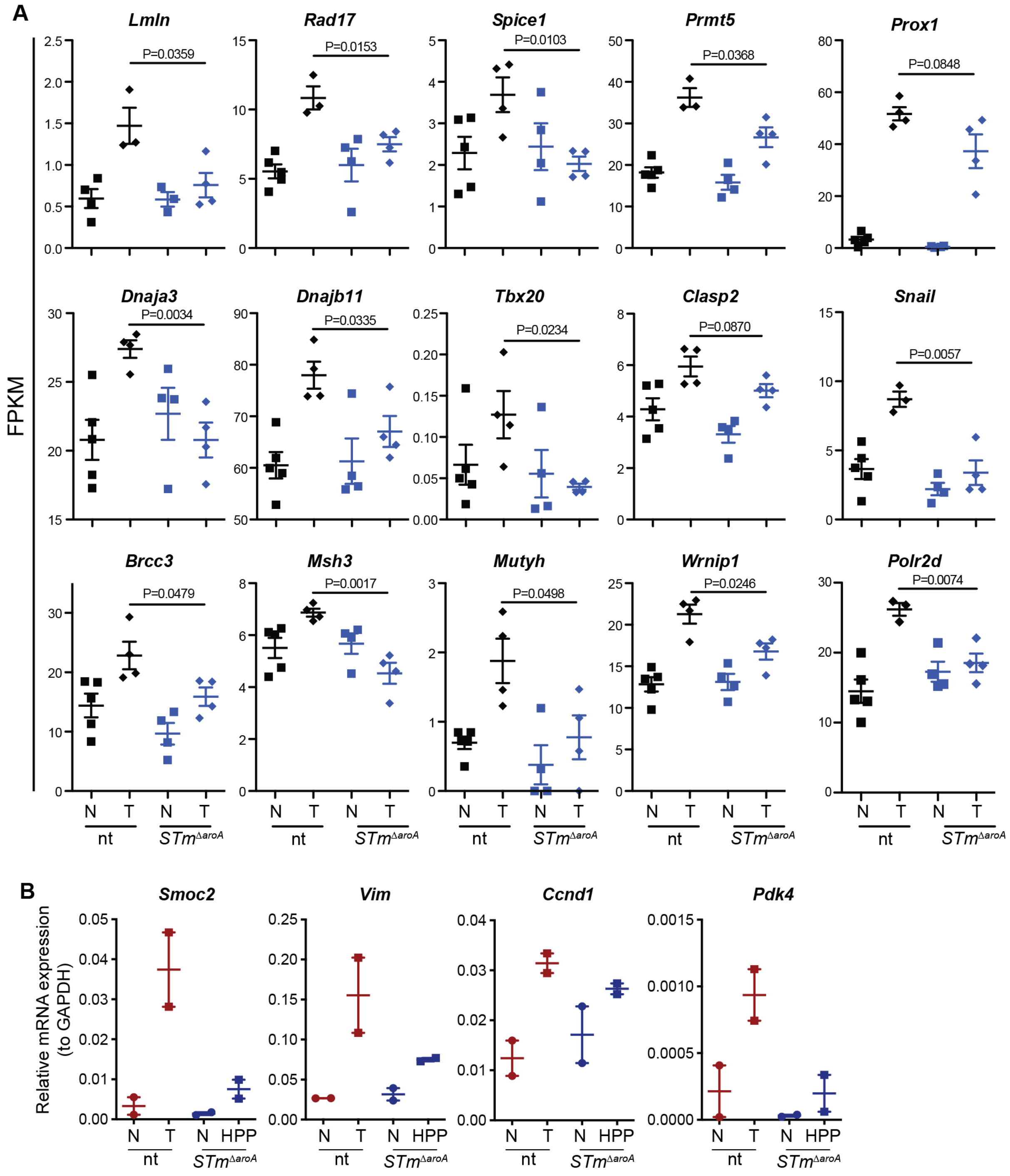

Figure S8: ***STm***$^{\Delta aroA}$ **treatment alters the transcriptional landscape of tumors.**
(**A**) Top differentially expressed genes (DEGs) identified by RNA sequencing. Tumors show an increased expression of a range of genes associated with processes from cell cycle regulation, DNA repair to epithelial-to-mesenchymal transition. These are reduced following treatment. (**B**) qPCR of indicated genes in normal (N), tumor (T) or hyperplasia (HPP) colon tissue from GF mice (as per Fig. S6). Each data point is tissue from one mouse, error bars show mean±SD.

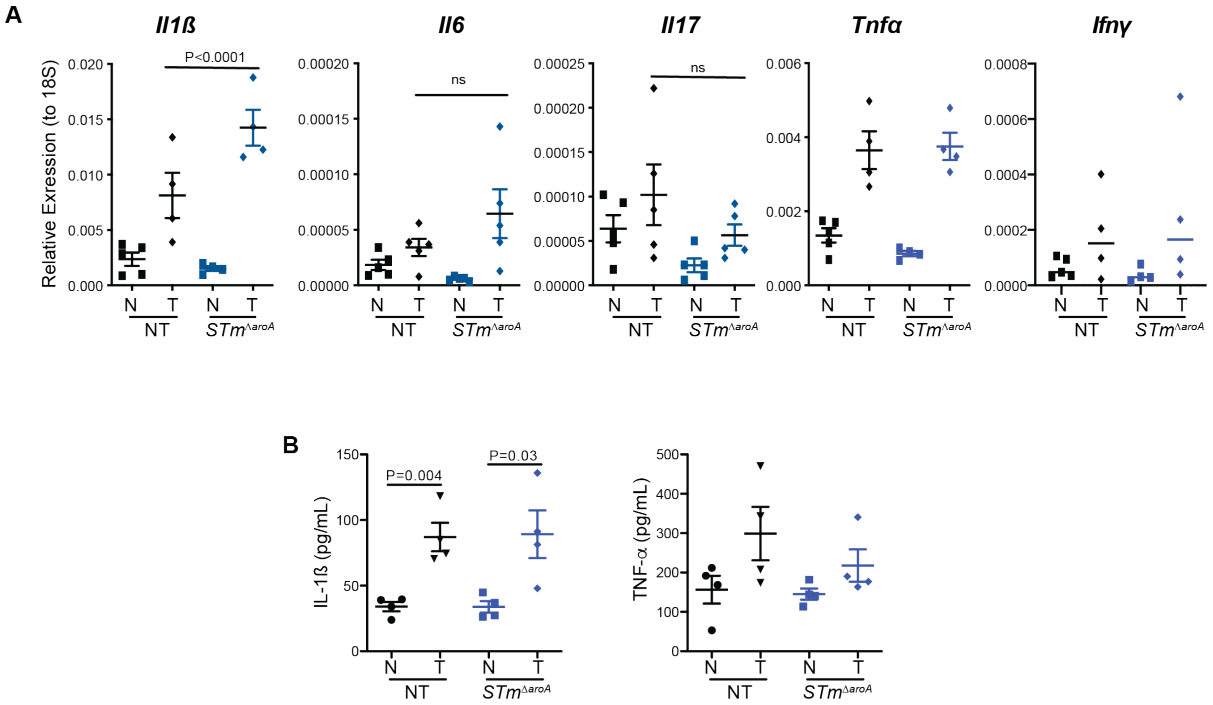

Figure S9: Cytokine levels following *STm*$^{\Delta aroA}$ treatment.
(**A**) qPCR for the indicated mRNAs from CAC-induced mice as per figure 1B. (**B**) Luminex analysis of protein extract from tumors of CAC-induced mice as per figure 1B. One-way ANOVA with post-hoc Turkey's test was used. Each data point is tissue from one mouse, error bars show mean±SD.

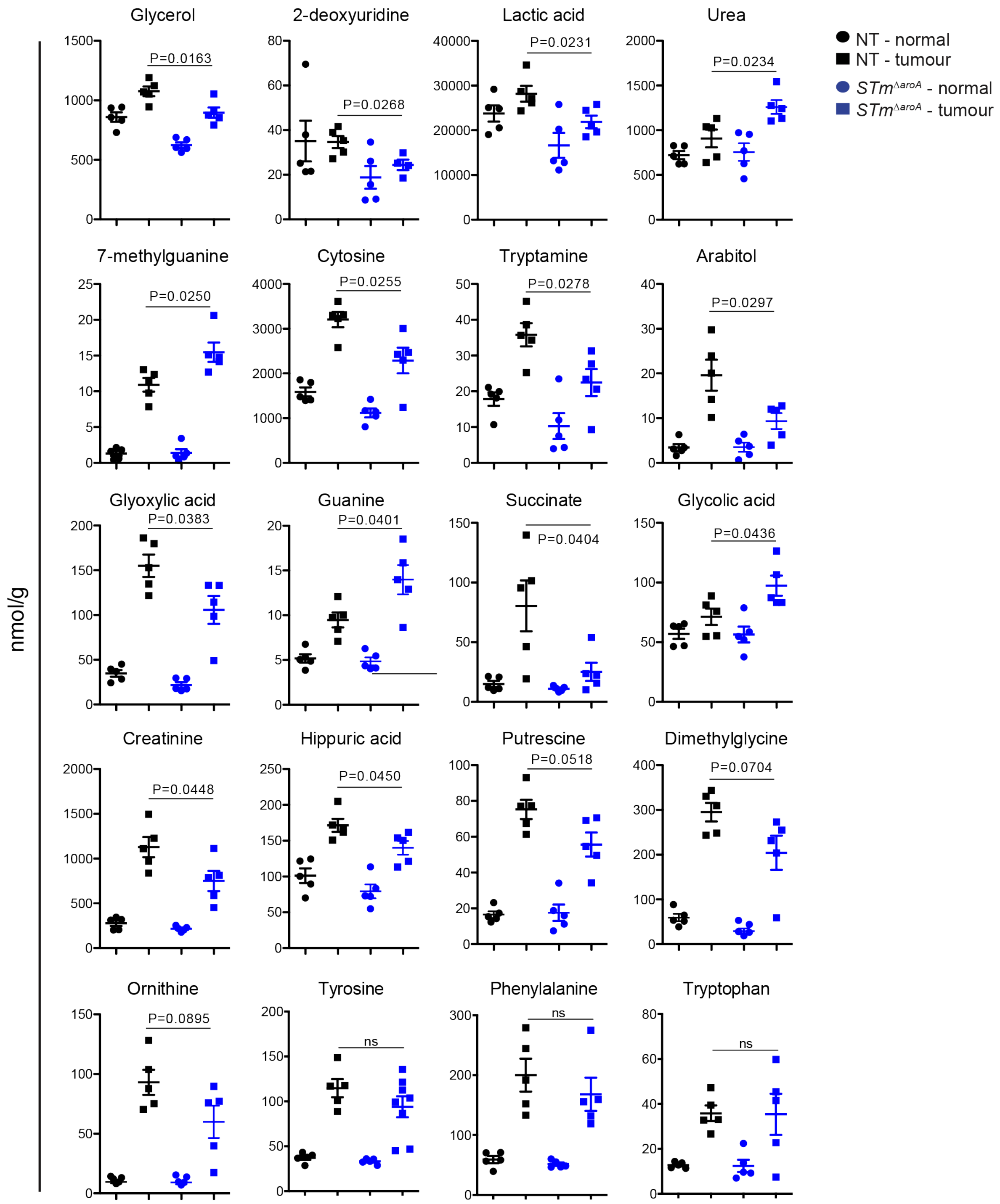

Figure S10: Metabolomics analysis after 6 weeks *STm*$^{\Delta aroA}$ treatment in vivo.
Top altered metabolites detected as described in Figure 5. One-way ANOVA with Bonferroni post-test. P-values comparing NT T and *STm*$^{\Delta aroA}$ T shown. Each data point is tissue from one mouse, error bars show mean±SD.

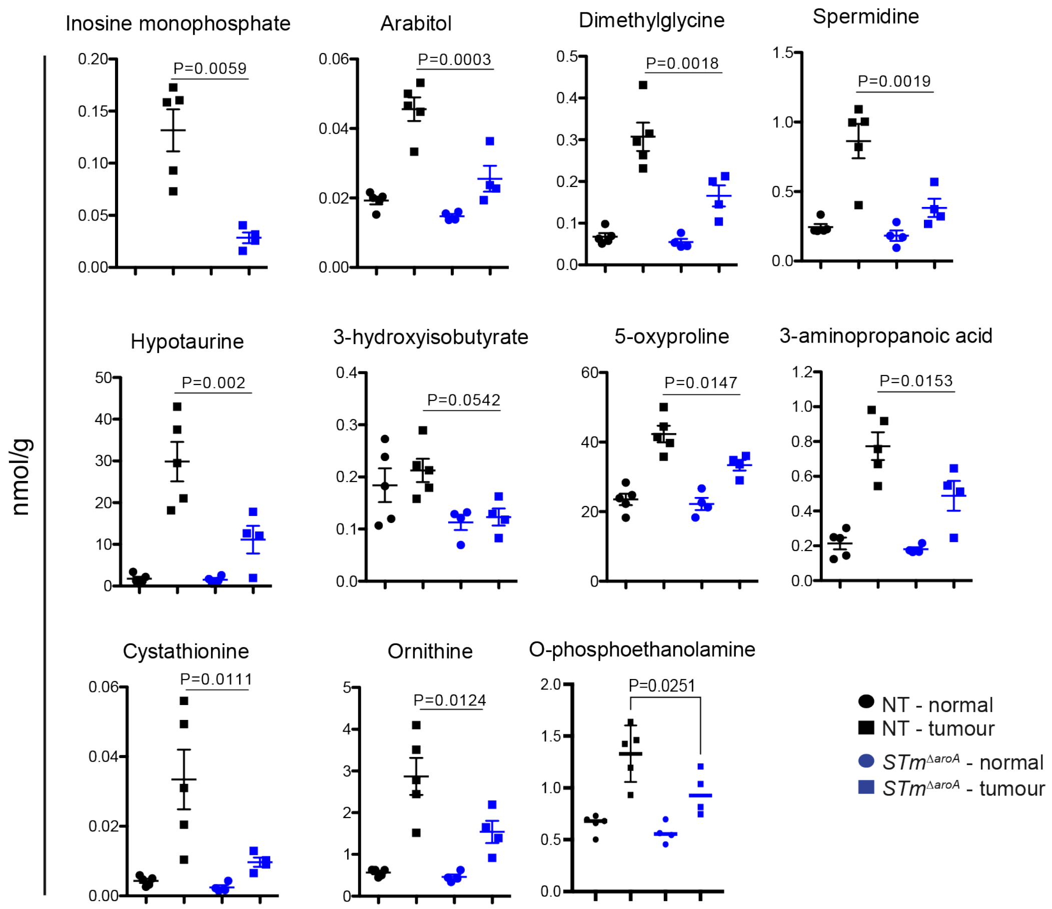

Figure S11: Metabolomics analysis after 24 hours *STm*$^{\Delta aroA}$ treatment in vivo.
Top altered metabolites detected as described in Figure 5. One-way ANOVA with Bonferroni post-test. P-values comparing NT T and *STm*$^{\Delta aroA}$ T shown. Each data point is tissue from one mouse, error bars show mean±SD.

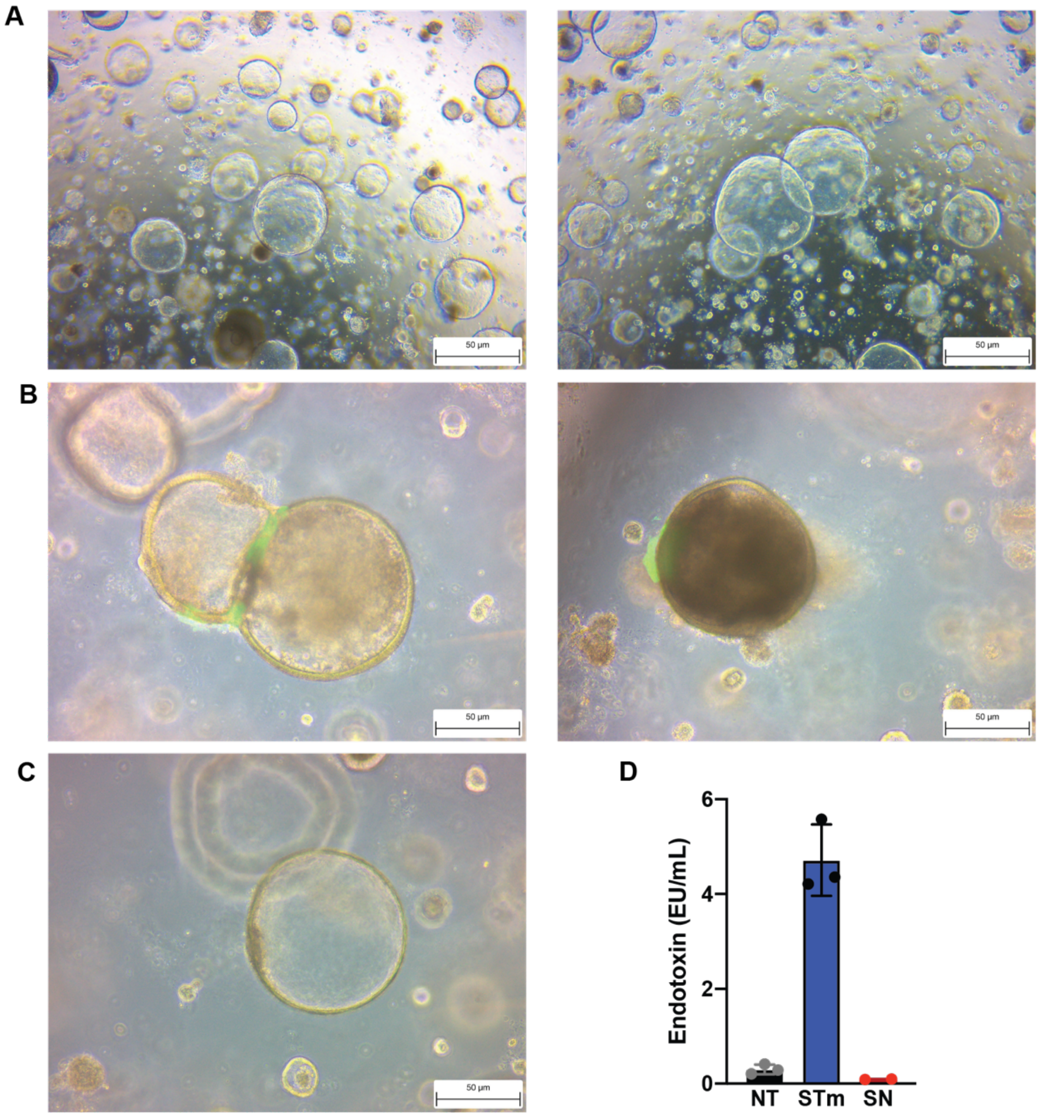

Figure S12: Tumor organoid morphology and infection.
(A) Tumor organoids are grown as indicated in methods. Representative images of *Apc*$^{min/+}$-derived SI tumor organoids at D5 post-split before infection. (**B**) 24 hours post-infection with GFP-expressing *STm*$^{\Delta aroA}$, brightfield overlaid with GFP, (**C**) 24 hours control treated , brightfield overlaid with GFP. (**D**) LAL assay for endotoxin in 10 kDa filtered SN from *STm*$^{\Delta aroA}$ culture used in Figure 6G and H.

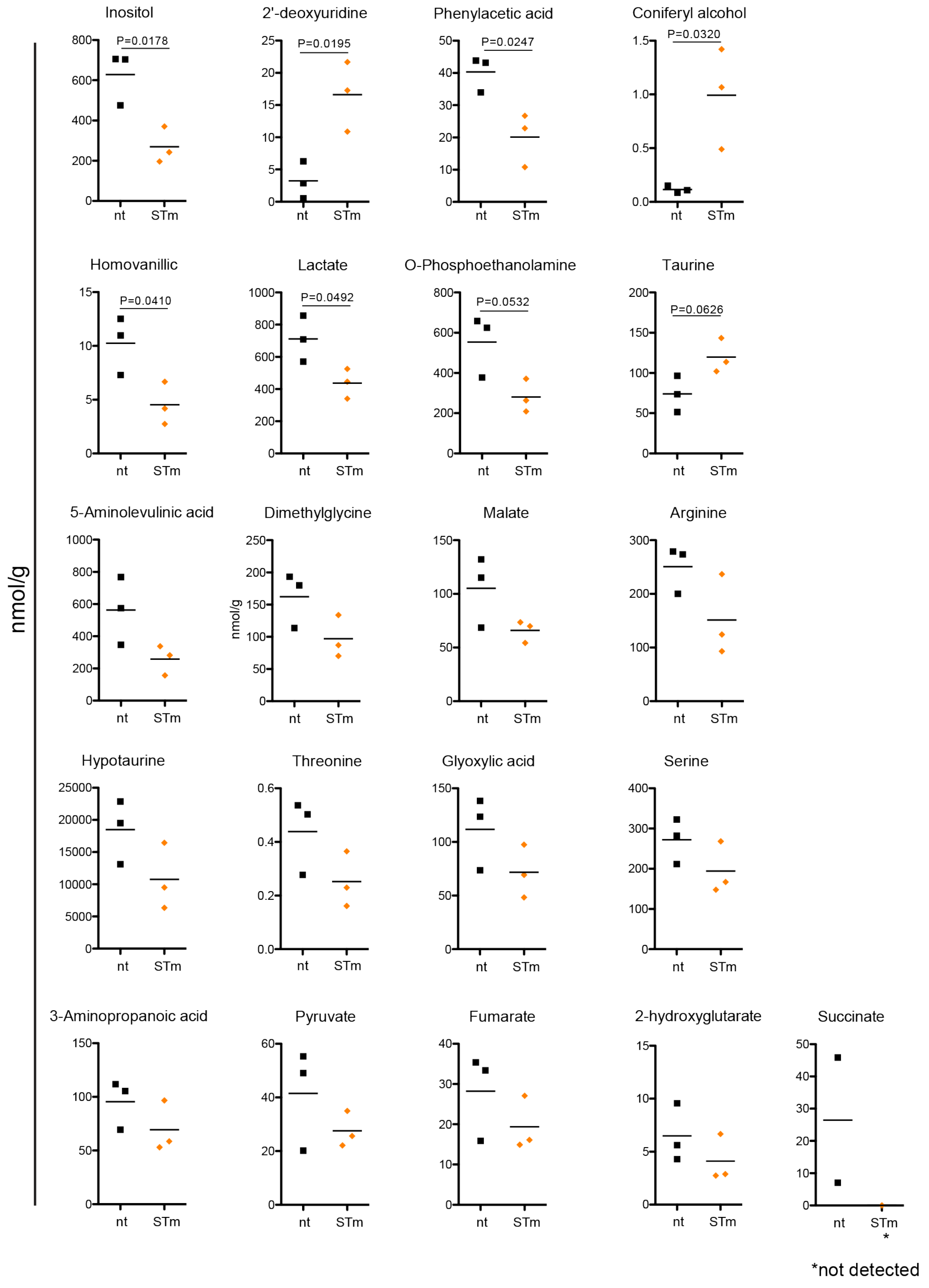

Figure S13: Metabolomics analysis after 24 hours *STm*$^{\Delta aroA}$ treatment in vitro. Top altered metabolites detected as described in Figure 6. Each point represents an independent well of organoids.

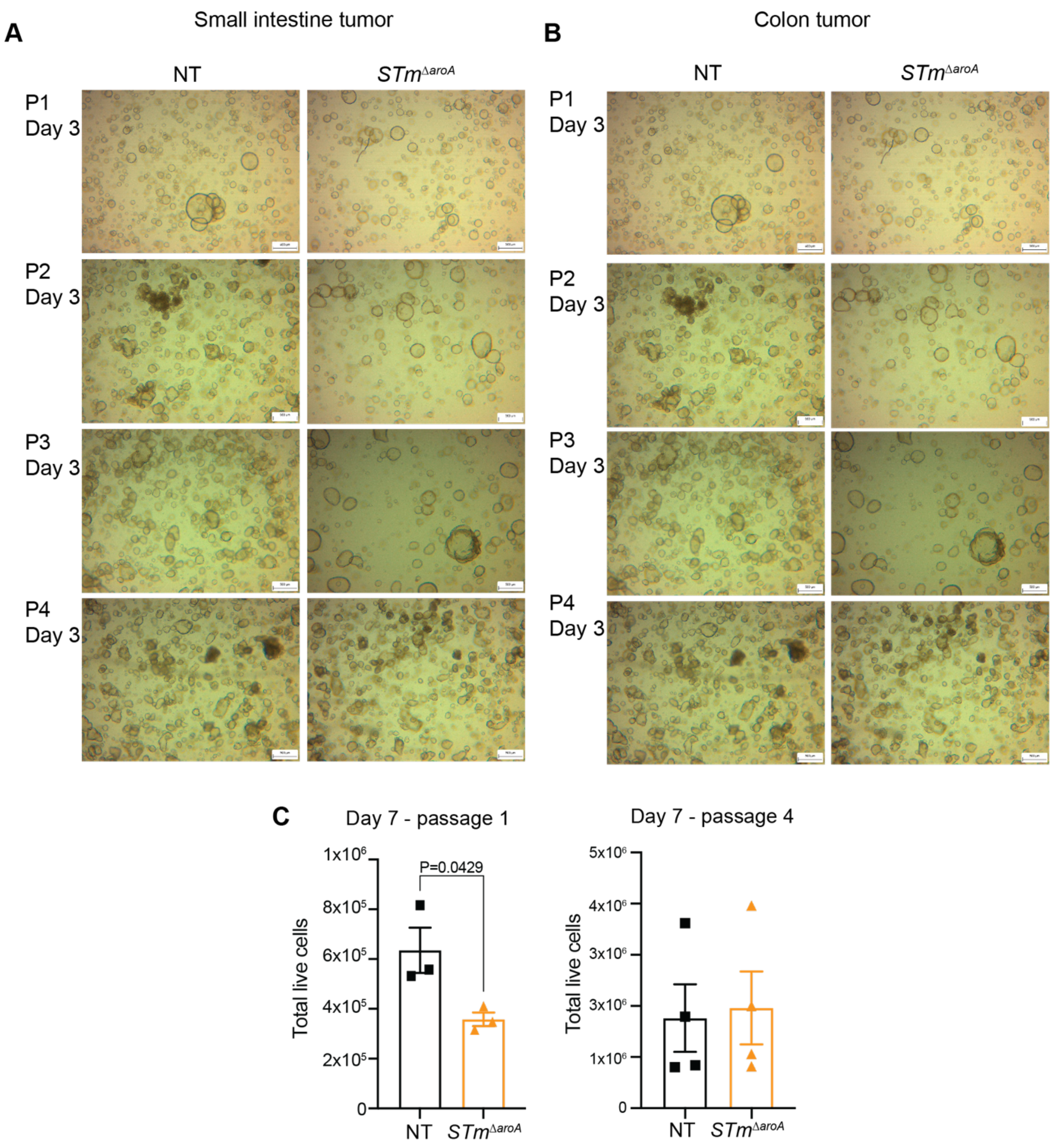

Figure S14: Long term passaging recovers *STm*$^{\Delta aroA}$ treated organoid growth.
SI (**A**) or Colon (**B**) tumor organoids (*Apc*$^{min/+}$-derived) were infected as described. After 24 hours they were dissociated into single cells, counted and seeded at equal density, then passaged at equal rations until the end pf 4 weeks (weekly passage). Representative images shown. (**C**) shows the cell number at the end of passage 1 and passage 4. Each dot represents an independent well. Two-way T-test. Data is representative of 3 experiments.

Table S3: List of metabolites with VIP score > 1 in tumors treated with *STm*$^{\Delta aroA}$ for 6 weeks.

Name	VIP score	fold change	P value
Glycerol	1.9801	0.8329	0.0163
2'-Deoxyuridine	1.9727	0.7054	0.0268
Lactic acid	1.9058	0.7767	0.0231
Urea	1.9031	1.3869	0.0234
7-Methylguanine	1.8888	1.4163	0.0250
Cytosine	1.8837	0.7140	0.0255
Tryptamine	1.8638	0.6275	0.0278
Arabitol	1.8477	0.4773	0.0297
Threonic acid	1.8113	1.5825	0.0345
2-Deoxy-glucose	1.7847	1.5725	0.0382
Glyoxylic acid	1.7838	0.6815	0.0383
Guanine	1.7717	1.4754	0.0401
Succinic acid	1.7699	0.3128	0.0404
Glycolic acid	1.7492	1.3657	0.0436
Creatinine	1.7423	0.6650	0.0448
Hippuric acid	1.7407	0.8170	0.0450
Putrescine	1.6135	0.7501	0.0691
Dimethylglycine	1.6076	0.6924	0.0704
3-Aminoisobutyric acid	1.5878	1.9660	0.0748
3-Aminopropanoic acid	1.5855	0.7546	0.0754
Xylose	1.5652	2.2137	0.0801
Ornithine	1.5272	0.6445	0.0895
5-Aminovaleric acid	1.5222	4.8578	0.0908
Indol-3-acetic acid	1.4590	1.6265	0.1081
Cellobiose	1.4068	1.6114	0.1238
Xanthosine	1.3765	1.7125	0.4535
3-Hydroxyglutaric acid	1.3594	0.8171	0.1393
Malic acid	1.3564	0.8042	0.1403
Glycyl-Glycine	1.3298	0.8518	0.1495
Sarcosine	1.3274	2.5237	0.1504
Allantoin	1.3171	1.3080	0.1540
Methionine	1.2888	1.6375	0.1644
Galactosamine	1.2835	0.7079	0.1664
Galactose	1.2715	0.7943	0.1710
Psicose	1.2256	0.7198	0.1891
Azelaic acid	1.2253	2.3617	0.1892
Norepinephrine	1.2137	1.4135	0.1940
Maltose	1.1786	0.6873	0.2087
Thymine	1.1628	0.7944	0.2156
O-Acetylserine	1.1543	0.7792	0.2193
Fucose	1.1515	1.5400	0.2206
Glucono-1,5-lactone	1.1514	0.8112	0.2206
Mannose	1.1222	0.8146	0.2338
Dihydrouracil	1.1212	0.3632	0.2527
Lyxose	1.1023	1.2183	0.2430
Lactitol	1.1004	0.1406	0.2469
3-Hydroxybutyric acid	1.0600	0.7720	0.2634
Fructose	1.0328	0.8219	0.2769
Phosphoric acid	1.0182	0.9116	0.2843
Hypotaurine	1.0104	0.6991	0.2883
2-Aminoadipic acid	1.0064	1.3342	0.2904

Table S4: List of metabolites with VIP score > 1 in tumors treated with *STm*$^{\Delta aroA}$ for 24 hours in vivo.

Metabolite	VIP score	fold change	P value
Inosine monophosphate	1.9117	0.2165	0.0031
Ribitol	1.9078	0.5208	0.0032
Cellobiose	1.8644	0.3229	0.0049
Arabitol	1.8548	0.5605	0.0054
Citric acid	1.8209	0.6098	0.0072
Trehalose	1.8013	0.3828	0.0084
Maltose	1.7993	0.4144	0.0085
Glucose	1.7758	0.4263	0.0101
Fumaric acid	1.7675	0.7185	0.0108
Isomaltose	1.7439	0.5221	0.0337
Dimethylglycine	1.7136	0.5386	0.0154
Lactulose	1.7134	0.6199	0.0155
Spermidine	1.7116	0.4437	0.0156
Hypotaurine	1.6867	0.3729	0.0182
3-Hydroxyisobutyric acid	1.6852	0.5794	0.0184
Lactitol	1.682	0.318	0.0187
5-Oxoproline	1.6626	0.7883	0.021
Xanthosine monophosphate	1.5912	0.624	0.0307
Xylitol	1.5887	0.4935	0.0311
Dihydroxyacetone phosphate	1.5866	0.6799	0.0314
Galactitol	1.5846	0.697	0.0317
Malic acid	1.582	0.7039	0.0321
3-Aminopropanoic acid	1.508	0.6303	0.0453
Cystathionine	1.5071	0.2898	0.0455
2-Hydroxyglutaric acid	1.5053	0.653	0.0459
Ornithine	1.4971	0.5365	0.0475
Cystine	1.4846	0.1547	0.041
Orotic acid	1.4598	0.5003	0.0555
O-Phosphoethanolamine	1.4504	0.7158	0.0576
Mannitol	1.4501	0.6358	0.0577
Succinic acid	1.4359	0.6271	0.061
Adenine	1.4174	0.6324	0.0656
Galactose	1.4101	0.6707	0.0674
Glucono-1,5-lactone	1.409	0.6451	0.0677
4-Hydroxyproline	1.4049	0.655	0.0688
Glucosamine	1.4024	1.54	0.0694
Glycine	1.3736	0.6654	0.0771
2,3-Bisphosphoglyceric acid	1.3672	1.6316	0.0789
Threonine	1.3359	0.7095	0.088
Glycerol 3-phosphate	1.3344	4.6072	0.0884
Cadaverine	1.3212	0.7518	0.0925
Glutamic acid	1.3211	0.7397	0.0925
Lactic acid	1.3108	0.8793	0.0956
Glycerol 2-phosphate	1.3081	1.7895	0.0966
Norvaline	1.3072	0.4694	0.0969
Cystamine	1.2993	0.5627	0.0994
Threonic acid	1.2797	0.764	0.1059
Lysine	1.2702	0.6183	0.1091
Phosphoenolpyruvic acid	1.2513	1.3313	0.2217
Tryptophan	1.2467	0.4	0.1173
Tyramine	1.242	0.6351	0.119
Arginine	1.242	0.6349	0.119
Ethylmalonic acid	1.2372	1.5596	0.1207
Erythrose 4-phosphate	1.2264	0.5996	0.1247

Ureidosuccinic acid	1.2199	0.7154	0.1271
Niacinamide	1.2189	0.8437	0.1275
Histidine	1.2025	0.6424	0.1338
Allantoin	1.1996	0.5463	0.1349
Proline	1.1976	0.736	0.1357
Ribonic acid	1.1963	0.736	0.1362
2-Aminoadipic acid	1.1784	0.6401	0.1433
Pyruvic acid	1.1765	0.8362	0.1441
6-Phosphogluconic acid	1.1526	0.4985	0.1539
Oleamide	1.1515	0.4691	0.1544
Hydroquinone	1.1409	0.67	0.1588
2-Aminoisobutyric acid	1.1385	1.9668	0.1599
N-Acetylserine	1.1154	0.7525	0.17
Norepinephrine	1.1102	1.2179	0.1723
Taurine	1.1045	0.8301	0.1748
Mannose 6-phosphate	1.1003	0.7004	0.1768
4-Aminobutyric acid	1.0925	0.789	0.1803
Glutamic acid 5-methylester	1.0873	1.3579	0.1827
Glucose 6-phosphate	1.0682	0.7218	0.1917
Quinolinic acid	1.0502	0.0916	0.2003
2-Phosphoglyceric acid	1.0498	1.6942	0.1907
Aspartic acid	1.0399	0.8244	0.2054
Allose	1.0192	0.7538	0.2158
Glucaric acid	1.0118	0.663	0.1895
N-Acetylneuraminic acid	1.005	0.827	0.2231

Table S5: List of metabolites with VIP score > 1 in tumor organoids treated with *STm*$^{\Delta aroA}$ for 24 hours.

Metabolite	VIP score	fold change	P value
Inositol	1.8382	0.4290	0.0178
2'-Deoxyuridine	1.8269	5.1418	0.0195
Phenylacetic acid	1.7964	0.4992	0.0247
Coniferyl alcohol	1.7576	8.5705	0.0320
Homovanillic acid	1.7155	0.4414	0.0410
Lactic acid	1.7002	0.6143	0.0492
O-Phosphoethanolamine	1.6650	0.5077	0.0532
Fucose	1.6605	3.1011	0.0543
Indol-3-acetic acid	1.6476	0.7028	0.0577
Taurine	1.6293	1.6209	0.0626
5-Aminolevulinic acid	1.5572	0.4599	0.0839
Uridine	1.5306	2.1723	0.1633
Lyxose	1.5002	0.5528	0.1026
Dimethylglycine	1.4973	0.5974	0.1036
Galactose	1.4862	2.2448	0.1075
Malic acid	1.4547	0.6259	0.1188
Arginine	1.4504	0.6035	0.1204
Ribonic acid	1.4259	0.2950	0.1295
4-Aminobenzoic acid	1.4205	0.5189	0.1316
Hypotaurine	1.4123	0.5822	0.1348
Threonic acid	1.4033	0.5742	0.1383
Mannitol	1.3841	3.2098	0.1437
Spermidine	1.3798	3.1311	0.1476
Glyoxylic acid	1.3191	0.6411	0.1729
Sebacic acid	1.3114	0.6055	0.1762
Guanine	1.2997	2.1673	0.1813
Homocysteine	1.2973	2.7287	0.1824
2-Aminoethanol	1.2938	0.7175	0.1839
Phosphoenolpyruvic acid	1.2811	1.5814	0.1895
Suberic acid	1.2801	0.3291	0.1900
Serine	1.2790	0.7142	0.1905
Pantothenic acid	1.2545	0.7354	0.2016
3-Aminopropanoic acid	1.1723	0.7260	0.2407
Xylose	1.1591	3.2897	0.2472
Alanine	1.1475	1.4680	0.2530
Arabitol	1.1097	3.7650	0.2723
Glucose	1.1063	3.0801	0.5069
Sorbitol	1.1018	3.1654	0.5090
Tyrosine	1.0957	2.4572	0.2795
Pyruvic acid	1.0729	0.6639	0.2915
Fumaric acid	1.0709	0.6866	0.2926
Dodecanedioic acid	1.0598	0.6416	0.2985
3-Hydroxypropionic acid	1.0470	0.6421	0.3054
2-Hydroxyglutaric acid	1.0444	0.6318	0.3068
Ribose	1.0413	1.6181	0.3085
Pyridoxamine	1.0286	2.3168	0.3154
Glutaric acid	1.0275	0.3534	0.3160
Ureidopropionic acid	1.0198	0.5835	0.3202
Dopa	1.0162	1.7613	0.3222
Adenine	1.0124	1.6859	0.3243
Hypoxanthine	1.0080	5.7728	0.3267
4-Aminobutyric acid	1.0006	1.7863	0.3308
